# Electroacupuncture Inhibits Atherosclerosis through Regulating Intestinal Flora and Host Metabolites in Rabbit

**DOI:** 10.1155/2020/5790275

**Published:** 2020-10-31

**Authors:** Yuping Shen, Ze-Dong Cheng, Yi-Guo Chen, Rui sun, Xian-De Ma, Guo-liang Hou, Rui Wang

**Affiliations:** ^1^Department of Acupuncture and Moxibustion, Liaoning University of Traditional Chinese Medicine, Shenyang, China; ^2^Liaoning University of Traditional Chinese Medicine, Ministry of Education Key Laboratory of Visceral Phenomenon Theory and Application in Traditional Chinese Medicine, Shenyang, China

## Abstract

**Methods:**

In this study, general rabbit conditions, vascular histology, metabolites, and intestinal flora structures were analyzed. Integrated analysis of metabolomics and 16S rRNA sequencing were performed. All the rabbits were randomly divided into four groups. The rabbit model of atherosclerosis was established. The histopathological change in the common carotid artery was assessed by HE staining and the structural change in the flora by 16S rRNA sequencing. HPLC-TOF-MS and Agilent MPP 12.1 were integrated to identify and screen out differential metabolites. Correlational analyses of every differential metabolite with intestinal flora were integrated on Omicshare platform.

**Results:**

Atherosclerotic rabbits showed obvious changes in general conditions, significant fibrous cap and necrotic center on carotid artery, abnormal intestinal bacteria structure, and metabolites levels. Electroacupuncture improved the conditions, reduced lipid deposition on the carotid artery wall, diversified intestinal flora, and normalized host metabolism. Integrated analysis showed that 149 altered metabolites were related to 22 intestinal flora, among which eight intestinal floras and 21 metabolites have relationships with atherosclerosis.

**Conclusion:**

Electroacupuncture can effectively reverse atherosclerosis through manipulating the structural feature of intestinal flora to influence the host metabolites. The possible mechanisms involved activating signal pathways through host metabolites or affecting the activity of cardiovascular-related enzymes, or regulating host lipid metabolism directly.

## 1. Introduction

Harboring microbes with metabolic functions, intestinal flora is critical to human health. Species spectrum analysis based on 16S rRNA sequencing has comprehensively explored the role of intestinal flora, mainly its structure, in disease development [1]. Besides, metabolomics has been increasingly applied to unravel the functions of human microbiota [2]. Metabolomic analysis focuses on the endogenous metabolites of a biological system, in a holistic view that also appears in Traditional Chinese Medicine (TCM) [3]. Recent years have seen its breakthrough in dealing with cardiovascular disease. These technologies enable us to study on the relationship between intestinal flora and chronic diseases.

In the recent decade, the relationship between intestinal flora and chronic cardiovascular diseases has become a hot topic. The incidence and mortality from atherosclerosis have declined in some countries [4]. It is clear that the intestinal flora can metabolize dietary L-carnitine and choline into trimethylamine, a substance further transformed into trimethylamine-N-oxide (TMAO) in the liver. TMAO can increase cholesterol deposition in vascular cells and accelerate the formation of foam cells and subsequent atherosclerosis plaques. However, atherosclerosis is caused by complex factors, and the relationships between metabolites and intestinal flora remain to be discovered. Therefore, in this research, we focused on the intestinal flora associated metabolites that participates in atherosclerosis.

Therapeutic methods for atherosclerosis vary. Acupuncture has emerged as a new clinical option for atherosclerosis in China. Some clinical studies showed that acupuncture can decrease carotid intimal thickness (IMT) and Crouse scores in phlegm dampness constitution carotid atherosclerosis with unique advantages [5]. Another clinical study showed that acupuncture combined with intravenous administration of alprostadil achieve better effect than simple intravenous administration of alprostadil for lower limb atherosclerosis of early diabetes mellitus [6]. In animal experiments, acupuncture has the same effect. A study showed that electroacupuncture at Quchi (LI-11), Zhongwan (CV-12), and Fenglong (ST-4) could regulate HDL-C, LDL-C, and atherosclerosis index [7]. Our previous studies [8] showed that electroacupuncture could reduce the atherosclerotic plaques, regulate the abnormal changes of blood lipid [9], and hemorhelogy in rabbits [10], and point stimulation can optimize the structure of intestinal flora [11]. A study has also found that electroacupuncture can modulate the status of intestinal flora [12]. Intestinal flora is known to have relationship with atheroslcoersis, but the mechanism needs more evidence. Therefore, we designed this research to illustrate how electroacupuncture regulates rabbit intestinal flora to reverse the development of atherosclerosis.

## 2. Materials and Methods

### 2.1. Animals and Ethical Approval

A total of 24 male New Zealand white rabbits (weighing 2.5 ± 0.5 kg, aged 2–3 months) were kept under standardized conditions (light from 6 am to 6 pm, temperature 18–20°C, relative humidity 50–70%), with free access to food and water. Rabbits were supplied by the Jinan Jinfeng Experimental Animal Company. The certification number is SCXK(Lu)2014-0006. All animal procedures were approved by the Local Ethical Review Panel of the Liaoning University of TCM (Ethical Inspection, No. 21000092017018). After experiment, animals were euthanized with air needle injection.

### 2.2. Reagents

High-fat diet, consisting of 1% cholesterol, 3% lard, 10% egg yolk powder, and 86% common ingredients, was processed into pellet feed by Qianmin Animal Feed Processing Center in Yuhong District, Shenyang, China. Other materials included atorvastatin calcium tablets (code: 9H0285991, Beijing Jialin Pharmaceutical Co., Ltd.); penicillin sodium (code: H13020657, North China Pharmaceutical Co., Ltd.); disposable sterile PTCA balloon dilatation catheter (2.0 × 15 mm, Beijing Demarc Co., Ltd.); needles (diameter 0.32 mm, length 15 mm, Suzhou Global Co., Ltd.); electroacupuncture apparatus (Suzhou medical products Co., Ltd.); HPLC-grade methanol (Merck, Darm-stadt, Germany); distilled water (Mill-Q ultrapure water machine, Millipore, USA); HPLC-grade formic acid (Tianjin Kermel Chemical Company, Tianjin, China). Pipettes, EP tubes, 10% urethane, heparin, injection bottles, pipette tips, and oil red O dye were provided by Liaoning University of the TCM laboratory.

### 2.3. Model Establishment

After one week of adaptive feeding, the rabbits were randomly divided into five groups: control group (normal rabbits, *n* = 6) and model group (atherosclerotic rabbits, *n* = 6), AC group (atherosclerotic rabbits + atorvastatin, *n* = 6), and EA group (atherosclerotic rabbits + electroacupuncture, *n* = 6). From beginning to the end, the control group was fed with standard diet without any treatment. In model, AC, and EA groups, after four week's high-fat diet (120 g per day), carotid artery balloon injury was made. Then, the high-fat diet was continued for another four weeks.

### 2.4. Balloon Injury

After 12 h of fasting and water deprivation, rabbits were anesthetized with 10% urethane 3 ml/kg through the ear vein. The skin was cut to expose subcutaneous tissue at midline of neck. The right carotid artery and the right external carotid artery were dissected. After injection of 200 *μ*/kg heparin at the ear vein, the right external carotid artery was blocked at distal end. The proximal end was separated, and then a small hole was made at the right external carotid artery. A 3.0 × 20 mm PTCA balloon catheter was inserted into the right carotid artery through a point about 4-5 cm to the right external carotid artery incision. Under 5-6 atmospheres, the saline was used to fill balloon. This process was repeated for three times and each time thirty seconds. The inserted catheter was pulled out, and the right external carotid artery was ligated. Meanwhile, intramuscular injection of 1,000,000 units of penicillin was administered after surgery for three days to prevent infection. After the balloon injury, the control group was fed with normal diet while the others with high-fat diet.

### 2.5. Experimental Treatment

After model establishment, atorvastatin calcium tablets (1 mg/kg/d) were added into high-fat diet in the AC group for four weeks. The EA group was treated with electroacupuncture therapy for four courses.

### 2.6. Electroacupuncture

Acupoints Neiguan (PC-6), Zusanli (ST-36), and Guanyuan (BL-26) were stimulated with sterile acupuncture needles. The needle was inserted by 0.2-0.3 cm for PC-6, 0.5–0.8 cm for ST-36, and 0.3–0.5 cm for BL-26. PC-6 was located in the gap of radius and ulna, on the line connecting the rasceta with the ulnar side of the biceps tendon, and 1/6 to the midpoint of the rasceta. ST-36 was located at 2/5 of the line connecting the navel and symphysis pubis. BL-26 was located at the upper 1/5 to the intersection of lateral calf, about 1.2 cm below the fibular head, 1 cm behind the posterior tibial crest. According to the rabbit's tolerance and local muscle twitching, the electroacupuncture apparatus was powered with rarefaction wave, 1 mA consuming current, and 2 Hz frequency. This therapy was performed once a day, 20 minutes per treatment, six days per course, and with an interval of one day after a course.

### 2.7. Sample Collection

At the end of the treatments, all groups were anesthetized with 10% urethan (3 ml/kg) through ear vein. Two milliliters of blood were drawn for metabolomics. After euthanasia, partial fragment of the right carotid artery and fresh feces were collected. The plasma samples and feces were stored in fridge at −80°C. Excess tissues of the right carotid arteries were eliminated and fixed with 10% neutral formalin.

### 2.8. General Conditions

The general conditions were graded and scored (Table 1). The scores of every rabbits in each group before and after the study were compared [13, 14].

### 2.9. Histopathology Analysis

#### 2.9.1. HE Staining

The right carotid artery was embedded by paraffin was transversely sectioned (thickness of 5 *μ*m, 4 sections per artery) and HE stained. After sealing, the pathological changes in arterial tissue were observed and photographed using a digital microscope.

#### 2.9.2. Red Oil Staining

After 24 h of fixation, parts of carotid arteries were washed by PBS water for 3 times. The carotid artery was cut longitudinally and immersed in the oil red dye for 1 h. The artery tissue was washed with 75% ethanol, until the fatty plaque turned red and other parts turned milky white. Then, the artery tissue was washed with distilled water for three times and fixed with needles.

### 2.10. Plasma Sample Preparation

All plasma samples were unfrozen at room temperature before HPLC-TOF-MS analysis. In HPLC-TOF-MS analysis, every 200 *μ*L of plasma was mixed with 600 *μ*L of methanol. After vortex-mixing for 2 min, the mixture was centrifuged (7,500 rpm, 15 min, 4°C) to precipitate the proteins. The supernatant was transited into an EP tube with a proper pipette and dried by nitrogen concentration. The samples were dissolved with 100 *μ*L of methanol and centrifuged again (2,000 rpm, 2 min, 4°C). Finally, 90 *μ*L of supernatant was transferred into an autosampler vials with a proper pipette.

### 2.11. Data Processing and Multivariate Analysis

The mass data were analyzed with Agilent Qualitative Analysis software, Mass Hunter Qualitative analysis software (MHQ, USA) within molecular feather extraction (MFE) and with Agilent MPP software (version 12.1, Agilent Corporation, USA) for alignment and normalization. Using PCA analysis, the metabolites with variable importance in the projection (fold change > 2) and the *P* values of one-way ANOVA (*P* < 0.01) were selected as potential biomarkers. The potential markers were identified by MPP software with ID Browsers and contrasted in available biochemical databases, KEGG and National center for biotechnology information (NCBI).

### 2.12. HPLC-TOF-MS Analysis

The Agilent 1290 HPLC system (Agilent Corporation, USA), Agilent Time of flight Mass Spectrometer equipped with electrospray ionization, and Agilent Poroshell 120 SB-C18 column (100 mm × 4.6 mm i.d., 2.7 *μ*m, Agilent Corp, USA) were used for *t* HPLC-MS analysis. The column temperature was kept at 35°C, the flow rate of the mobile phase at 0.4 ml/min, and the injection volume at 4 *μ*L. Mobile phase consisted of 0.1% formic acid in water (A) and methanol (B). The column was eluted with 100% B first, then 20% A and 80% B for 10.00 min, and 100% B for 20.00 min. The parameters for HPLC-TOF-MS analysis were set as follows: ion source of: − ESI, capillary voltage of 3500 V, gas temperature of 250°C, drying gas of 9 L/min, nebulizer of 45 psig, and fragmentor voltage of 125 V. An injection with a needle of automatic-washing mode was employed on the autosampler.

### 2.13. Analysis of Intestinal Flora Structure and Multiomics Union

16S rRNA sequencing was performed by Shanghai Personal Biotechnology Co., Ltd. Multiomics union analysis was performed by Guangzhou Genedenovo Biotechnology Co., Ltd.

## 3. Results

### 3.1. General Condition Score

**Figure 1:**
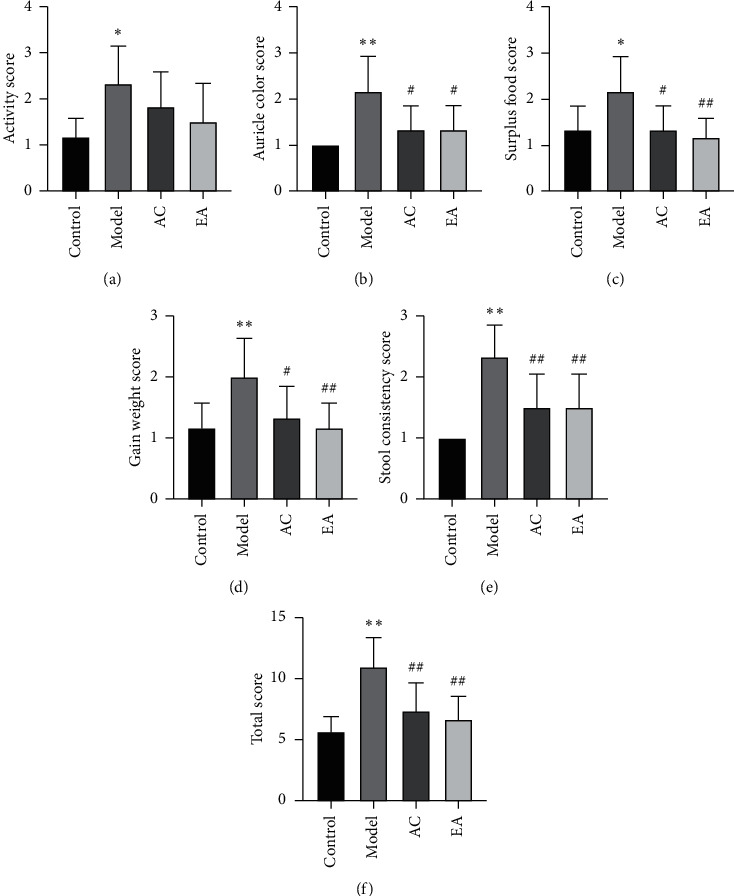
General condition scores. ^∗^*P* < 0.05 vs. control, ^∗∗^*P* < 0.01 vs. control. ^#^*P* < 0.05 vs. model, and ^##^*P* < 0.01 vs. model.

During the first week, rabbits were in good condition, and there was no significant difference between groups (*P* > 0.05). However, after modeling, the rabbits gradually showed decreased activity, loss of appetite, diarrhea, dark ear color, and general body lightening. But, the general condition of the rabbits treated with atorvastatin or electroacupuncture was better. The general condition score is shown in Figure 1.

### 3.2. Histopathological Results

#### 3.2.1. HE Staining Results

**Figure 2:**
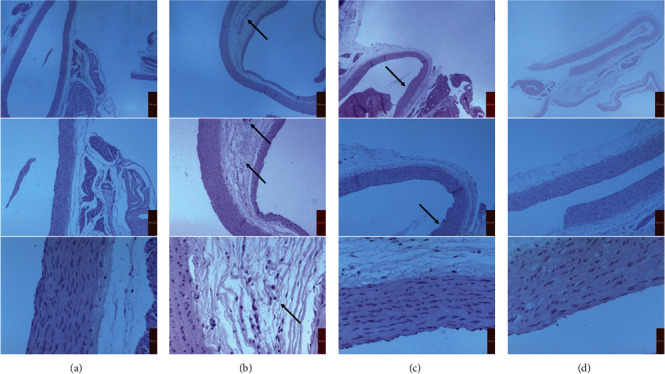
HE staining of atherosclerotic plaques (×40, ×100, and ×400). (a) Control group. (b) Model group. (c) AC group. (d) EA group.

The aorta tissues (Figure 2) in the control group showed uniform vascular, integrated endothelium, smooth vascular wall without lipid deposition, no foam cell accumulation, and atherosclerotic plaques. In contrast, the tissues in the model group showed obvious fibrous cap and necrotic center, indicating that the atherosclerotic models had been successful established. In AC and EA groups, the thickness of the vascular wall, the level of inflammatory cells, the infiltration of foam cells, and the plaque area decreased after atorvastatin or electroacupuncture.

#### 3.2.2. Red Oil Staining Results

**Figure 3:**
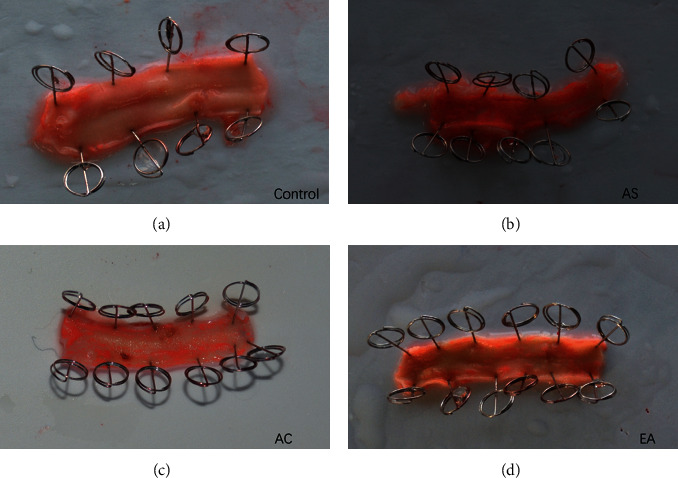
Oil red O staining of atherosclerotic plaque size.

Red oil staining results were showed the changes of atherosclerosis plaque size. After staining, the normal carotid artery tissue in the control group was milky white, while in the model group the lipid deposited on the endothelium, showing orange red (Figure 3). The orange red areas in the AC and EA groups were smaller than those in the model group.

### 3.3. Intestinal Flora Structure

**Figure 4:**
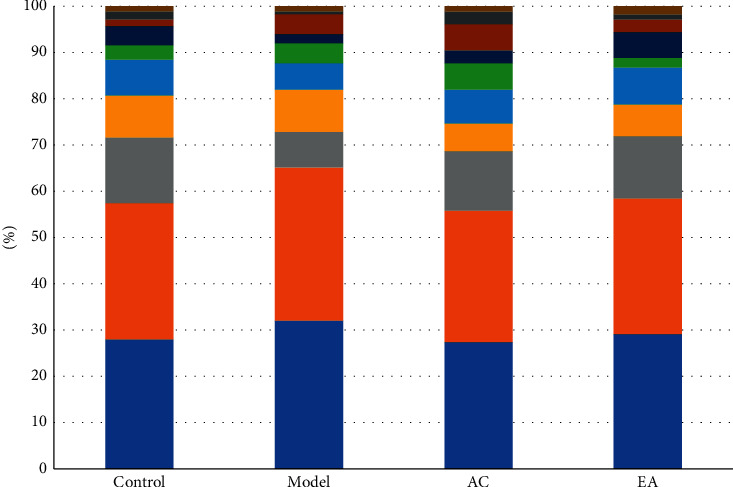
Intestinal flora abundance on family level.

The top ten most abundant of intestinal flora were visualized. Different color means different flora, and the length of each color column means the abundance of corresponding flora. The results showed that, on family level, the abundances of intestinal flora in different groups were different (Figure 4). ACE and Simpson indexes were used to show abundance and diversity (Table 2).

### 3.4. Metabolic Analysis Results

**Figure 5:**
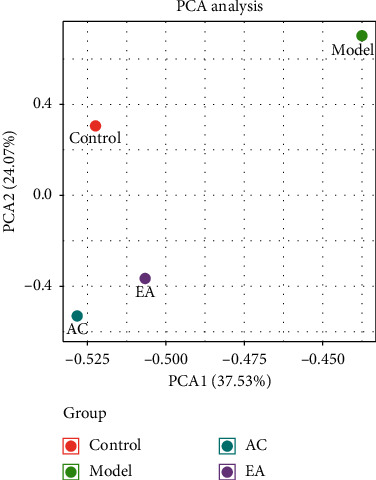
PCA of metabolites in different groups.

Low-molecular weight metabolites were separated completely in 20 min. The results of ANOVA using MPP software and principal component analysis (PCA) are shown in Figure 5. Obviously, the metabolites in different groups showed differences. The dimensional patterns in AC and EA groups were closer to that in the control group, which meant the metabolites in these three groups are similar. Actually, 222 metabolites showed statistic difference. Compared with control group, there were 70 metabolites upregulated and 151 metabolites downregulated. Compared with the model group, 117 metabolites in AC group and 84 metabolites in EA group were upregulated, while 86 metabolites in AC group and 136 metabolites in EA group were downregulated. The 132 metabolites in AC group and 129 metabolites in EA group showed the same tendency with control group, which indicated that electroacupuncture not only regulated the structure of intestinal flora but also the production of host metabolites.

### 3.5. Integration of Metabolomics and 16S rRNA Sequencing

**Figure 6:**
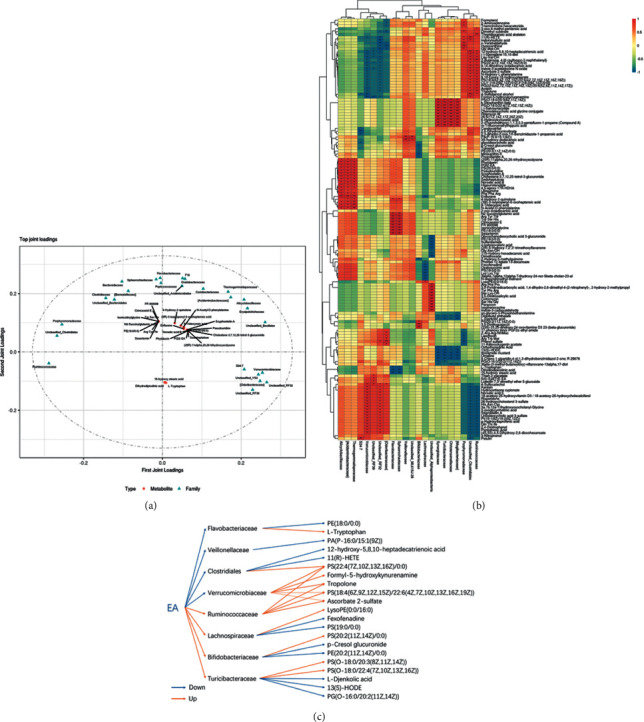
(a) Integrated metabolomics and 16S rRNA sequencing. ^∗^*P* < 0.05; ^∗∗^*P* < 0.01. (b) O2PLS analysis. (c) Possible methods.

The results of O2PLS analysis are shown in the Figure 6(a). The dots represent bacterium or metabolites. The greater the absolute value in the coordinates, the greater the degree of association between this element and another omics. Among the 222 metabolites with differential levels, 149 metabolites showed relationships with 22 intestinal flora (*P* < 0.05) (Figure 6(b)). According to the *Method for diagnosing heart disease through bacterial metagenomic analysis* [15], the changes of Thermogemmatisporaceae, Flavobacteriaceae, Verrucomicrobiaceae, S24-7, Lachnopiraceae, Bifidobacteriaceae, Turicibacteraceae, Veillonellaceae, Ruminococcaceae, and Clostridiales could be used in the diagnosis of cardiovascular disease, including atherosclerosis. These 10 families of intestinal bacterium were definitely related to 45 metabolites. According to KEGG, Lipid MAP, METLIN, and HMDB databases, 21 metabolites have been confirmed to be associated with the development of atherosclerosis. In this study, electroacupuncture regulated the 8 intestinal bacteria to influence 21 host metabolites to reverse atherosclerosis (Figure 6(c)).

## 4. Discussion

TCM holds that atherosclerosis develops with the stagnant *qi* and blood, usually caused by vascular phlegm and stasis. At present, the mechanism of atherosclerosis is not fully understood, but it is certain that the occurrence and development of atherosclerosis are closely related to the abnormal metabolic function of organism. The intestinal flora parasitizes in the intestines of animals and participates in the metabolic processes of the host. Intestinal flora has become a new therapeutic target for various metabolic diseases [16].

In previous study, three relevant findings that can support the use of electroacupuncture as an intervention to treat atherosclerosis. Firstly, point stimulation could change the structural features of intestinal flora [11]. Secondly, electroacupuncture has a good regulatory effect on myocardial ischemia diseases [17]. Thirdly, electroacupuncture could increase the concentration of drugs in specific viscera, which means electroacupuncture combined with drugs will achieve synergistic effect and improve drug efficacy. Besides, it has shown that stimulating PC-6 can benefit the coronary blood flow [18], stimulating ST-36 can decrease blood lipid [19] and stimulating BL-36 can optimize the structure of intestinal flora [20]. This is the mainly reason that why we choose electroacupuncture as the intervention in this study.

### 4.1. General Condition and TCM

According TCM theory, atherosclerosis is caused by the combination of phlegm, blood stasis, and toxin. In human body, atherosclerosis will develop with some signs and symptoms, like chest tightness, asthma, fatigue, obesity, or emaciation. In rabbits, fur, ear color, activity, weight gain, and feces condition are important indices, for example, the auricle color could be related with blood stasis. In this study, those symptoms worsen after modeling and relieve after treatment.

### 4.2. Changes in Histopathology

In this study, electroacupuncture reduced atherosclerosis plaque in EA groups. Favorable morphological changes confirmed the antiatherosclerotic effect of electroacupuncture. This incidence verified the antiatherosclerotic effect of electroacupuncture for atherosclerosis qualitatively.

### 4.3. Structural Feature Changes in Intestinal Flora by Electroacupuncture

Normal intestinal flora maintains homeostasis. Drug may decrease the probiotics, enrich the pathogenic bacteria or opportunistic bacterium [21], and overturn the structural features of intestinal flora [22]. In this study, the differences of the structural features of intestinal flora were obvious, suggesting that the occurrence of atherosclerosis may be related to abnormal structural features of the intestinal flora, and electroacupuncture can regulate this state.

### 4.4. Metabolites in Plasma Were Different between Groups

In the present study, 16S rRNA sequencing showed that either of atherosclerosis, atorvastatin application, and electroacupuncture could change intestinal flora structural features. In order to determine whether this change has an impact on the host, this study selected nontargeted metabonomics to test plasma metabolites. Our results showed that either of atherosclerosis lesions, atorvastatin application, and electroacupuncture could change plasma metabolism levels among the groups. The PCA patterns in model and control groups were different, which indicated that atherosclerosis had bad effect on the metabolism of the body. Interestingly, the PCA pattern in the EA group was different to that in model group, but similar to that in the control group, which demonstrates that electroacupuncture could have some therapeutic effect on this metabolic abnormality. And, some of 222 differentially produced metabolites were directly related to atherosclerosis: Ascorbate 2-sulfate [23, 24], hypoxanthine [25], Hippurate [26], 13(S)-HODE [27], inositol [28], 6-keto-PGF1*α* [29], N- acetylneuraminic acid [30], ubiquinone [31], glucuronide [32], L-tryptophan [33], 15(S)-HETE [34], etc. However, the current evidence is still unable to confirm whether these metabolic changes were caused by intestinal flora or not, so it was necessary to perform integrated analysis of two omics.

### 4.5. Almost 67% Differential Metabolites Were Related to Intestinal Flora

The integrated analysis of omics showed that 67% of 222 differential plasma metabolites were closely related to 22 intestinal flora, suggesting that intestinal flora is not only an important participant in the development of atherosclerosis but also a medium for electroacupuncture to treat atherosclerosis.

The host metabolites can be influenced by electroacupuncture through intestinal flora to activate some signal pathway. Taking Turicibacteriaceae as an example, electroacupuncture can increase its abundance in atherosclerotic rabbits, decrease the metabolites 13(S)-HODE and L-Djenkolic acid in plasma, and then eliminate the atherosclerotic plaque. L-djenkolic acid [35] could produce oxygen ion to trigger low-density lipoprotein oxidation reaction, which will accelerate the formation of atherosclerotic plaque. 13(S)-HODE is a kind of trans-unsaturated fatty acid, which is harmful to the cardiovascular system; however, the fact is that 13(S)-HODE is negatively correlated with Turicibacteriaceae, but its level in the plasma is high. The reason may be that one metabolite may be affected by multiple intestinal flora. 13(S)-HODE is also affected by Christensenellaceae and (Mogibacteriaceae). 13(S)-HODE is a ligand in the PPAR signal pathway existing in adipocytes. Peroxisome proliferator-activated receptors (PPARs) are nuclear hormone receptors that can be activated by fatty acids and their derivatives, including three major members: PPAR*α*, PPAR*β*/*δ*, and PPAR*γ*. A lot of evidence showed that atorvastatin lowers lipid level through activating PPARs. This is why 13(S)-HODE is higher after electroacupuncture. Moreover, we can speculate that electroacupuncture may also relieve atherosclerosis by employing the intestinal flora to regulate the levels of host metabolites and then activate PPARs.

Host metabolites can be affected by electroacupuncture through intestinal flora to adjust the activity of cardiovascular-related enzymes, Tropolone, for instance, a kind of catechol-O-methyltransferase (COMT) inhibitor. COMT has been implicated in both depression and cardiovascular disease (CVD). A study showed that high COMT activity genotype Val158Met, associated with activity of COMT, can increase the risk of CVD [36]. Moreover, Hinokitiol, a derivative of Tropolone, can mediate p-JNK and p-PLC*γ*1 signaling pathways to achieve antiatherosclerotic plaque effects [37]. In this study, electroacupuncture can upregulate Ruminococcaceae and downregulate Verrucomicrobiaceae to upregulate Tropolone in plasma, which indicated that electroacupuncture may inhibit the activity of COMT by upregulating Tropolone through intestinal flora to prevent CVD.

Electroacupuncture also can influence host lipids through intestinal flora to adjust lipid metabolism, such as PS(22 : 4(7Z, 10Z, 13Z, 16Z)/0 : 0), LysoPE(0 : 0/16 : 0), PA(P-16 : 0/15 : 1(9Z)), PS(O-18 : 0/20 : 3(8Z, 11Z, 14Z)), PS(O-18 : 0/22 : 4(7Z, 10Z, 13Z, 16Z)), and PG(O-16 : 0/20 : 2(11Z, 14Z)). They belong to glycerophospholipid category, and electroacupuncture can adjust them through intestinal flora.

This study has some limitations. Firstly, the range of the mass-to-charge ratio that can be predicted by HPLC/ESI-TOF-MS is 100 to 1500, so beyond this range, molecular information cannot be captured. Secondly, the metabolomics in this study is nontargeted and metabolites cannot be accurately quantified. Therefore, the results of this study need to be verified by more experiments.

## 5. Conclusion

Electroacupuncture can effectively reverse atherosclerosis through manipulating the structural feature of intestinal flora to influence the host metabolites. The possible mechanisms involved activating the signal pathways through metabolites, or affecting the activity of cardiovascular-related enzymes, or regulating host lipid metabolism directly.

## Figures and Tables

**Table 1:** Scores of conditions.

Score	Activity	Auricle color	Surplus food (g)	Weight	Stool consistency
1	Active move, lively	Ruddy	<60	Gained >0.8 kg	Round and smooth
2	Active move, not lively	Pale	60–100	Gained <0.8 kg	Diarrhea, amount less than 10 ml
3	Inactive	Gloomy	>100	Lose weight	Diarrhea, amount more than 10 ml

**Table 2:** Alpha index of intestinal flora. ^∗^*P* < 0.05 vs. control, ^∗∗^*P* < 0.01 vs. control, and ^##^*P* < 0.05 vs. model.

Group	ACE	Simpson
Control	1466.1383 ± 165.9871	0.9826 ± 0.0066
Model	1050.4783 ± 141.9394^∗^	0.9548 ± 0.0276^∗∗^
AC	1305.4950 ± 134.5323^#^	0.9789 ± 0.0042^#^
EA	1308.0183 ± 75.8283^#^	0.9768 ± 0.0082^#^

## Data Availability

The data used to support the article are available in the article.

## Supplementary Materials

Supplementary Materials All data that involved in this manuscript have been uploaded.
