# Supplementary figures and images for: Electroacupuncture Inhibits Atherosclerosis through Regulating Intestinal Flora and Host Metabolites in Rabbit
Source: Evid Based Complement Alternat Med. 2020 Oct 31;2020:5790275. doi: 10.1155/2020/5790275 (PMC7676925; doi:10.1155/2020/5790275)

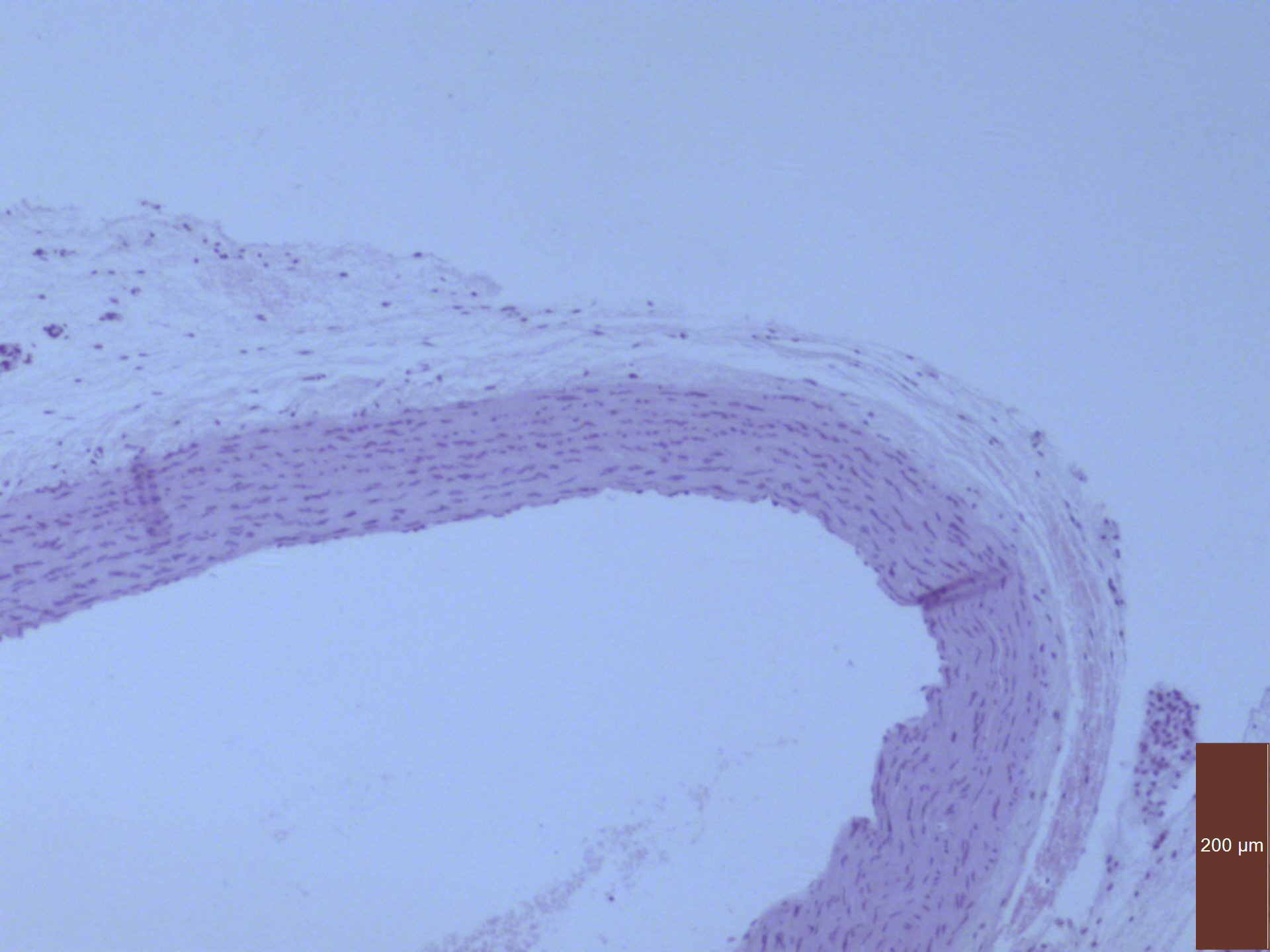

Supplement: Supplementary Materials — All data that involved in this manuscript have been uploaded. [file 5790275.f1.zip › 5790275.f1/AC group-100.jpg]

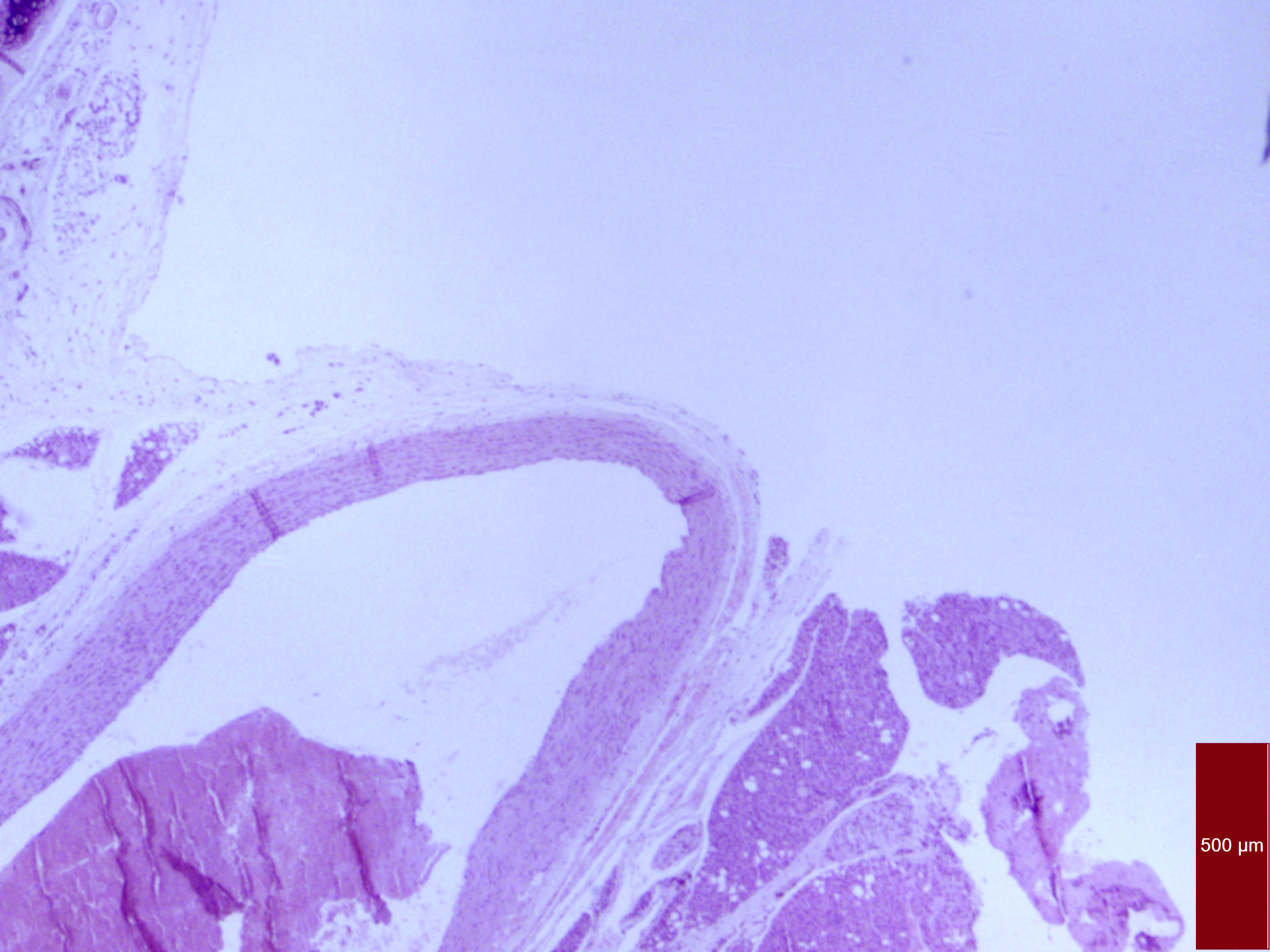

Supplement: Supplementary Materials — All data that involved in this manuscript have been uploaded. [file 5790275.f1.zip › 5790275.f1/AC group-40.jpg]

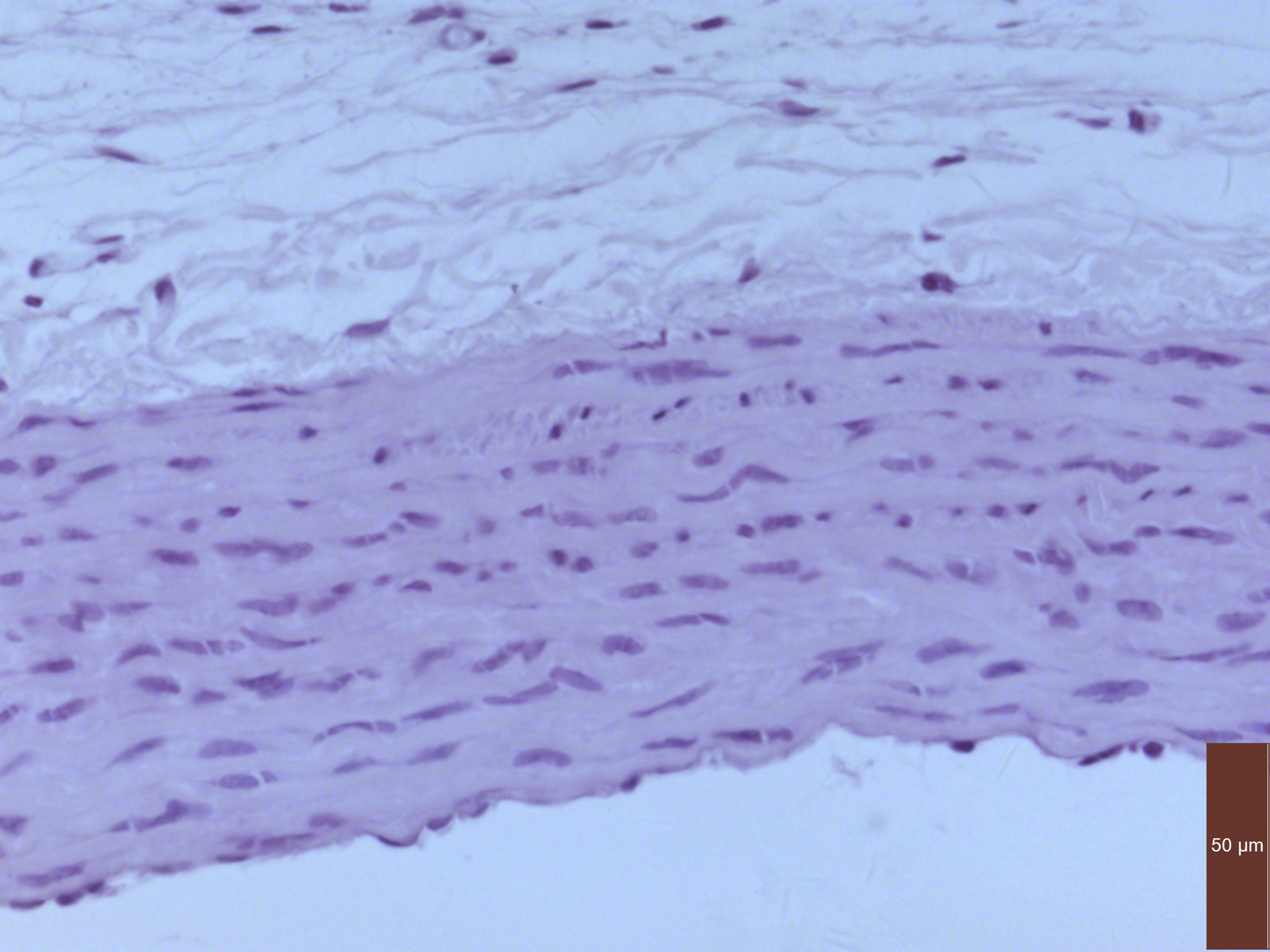

Supplement: Supplementary Materials — All data that involved in this manuscript have been uploaded. [file 5790275.f1.zip › 5790275.f1/AC group-400.jpg]

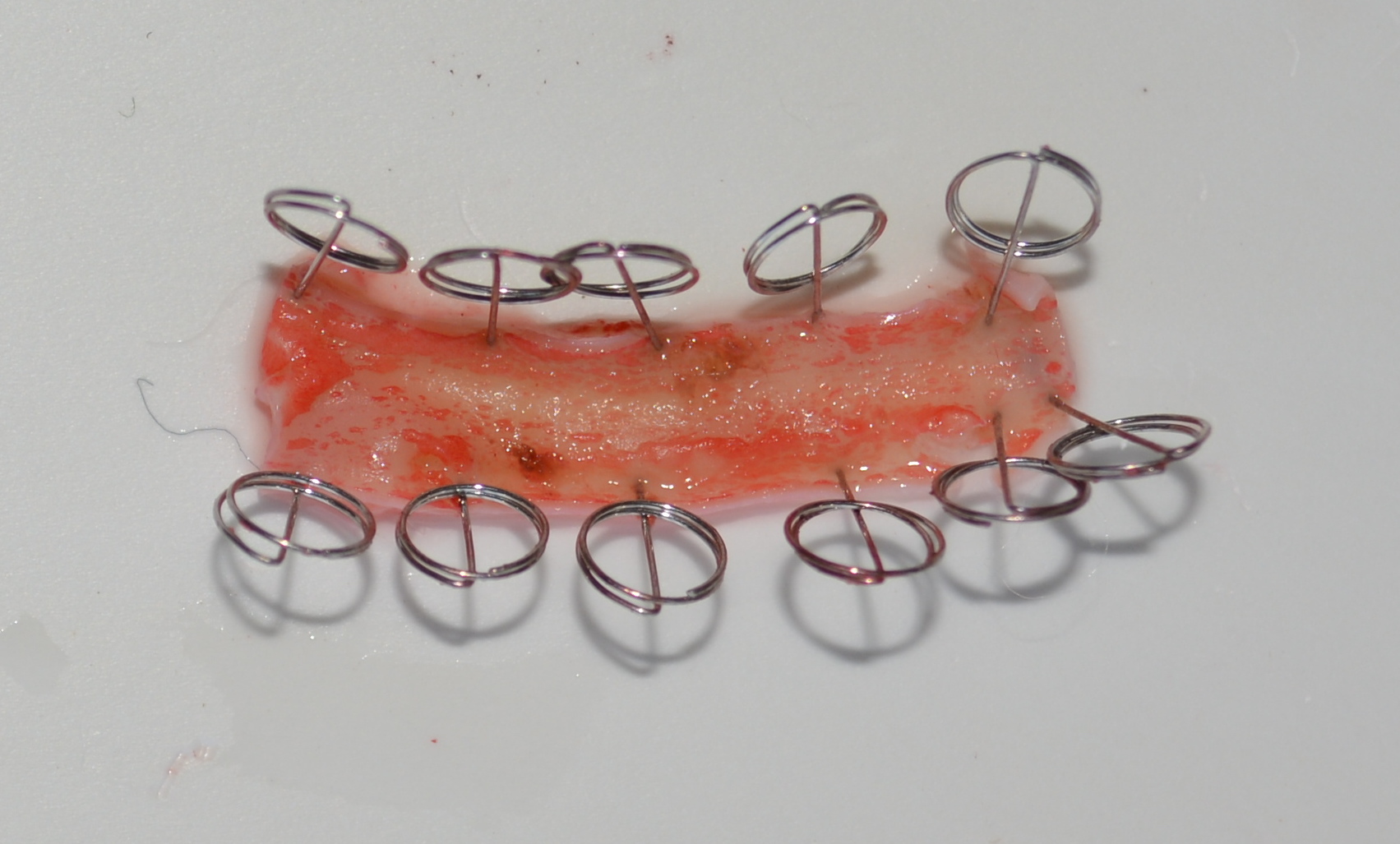

Supplement: Supplementary Materials — All data that involved in this manuscript have been uploaded. [file 5790275.f1.zip › 5790275.f1/AC.JPG]

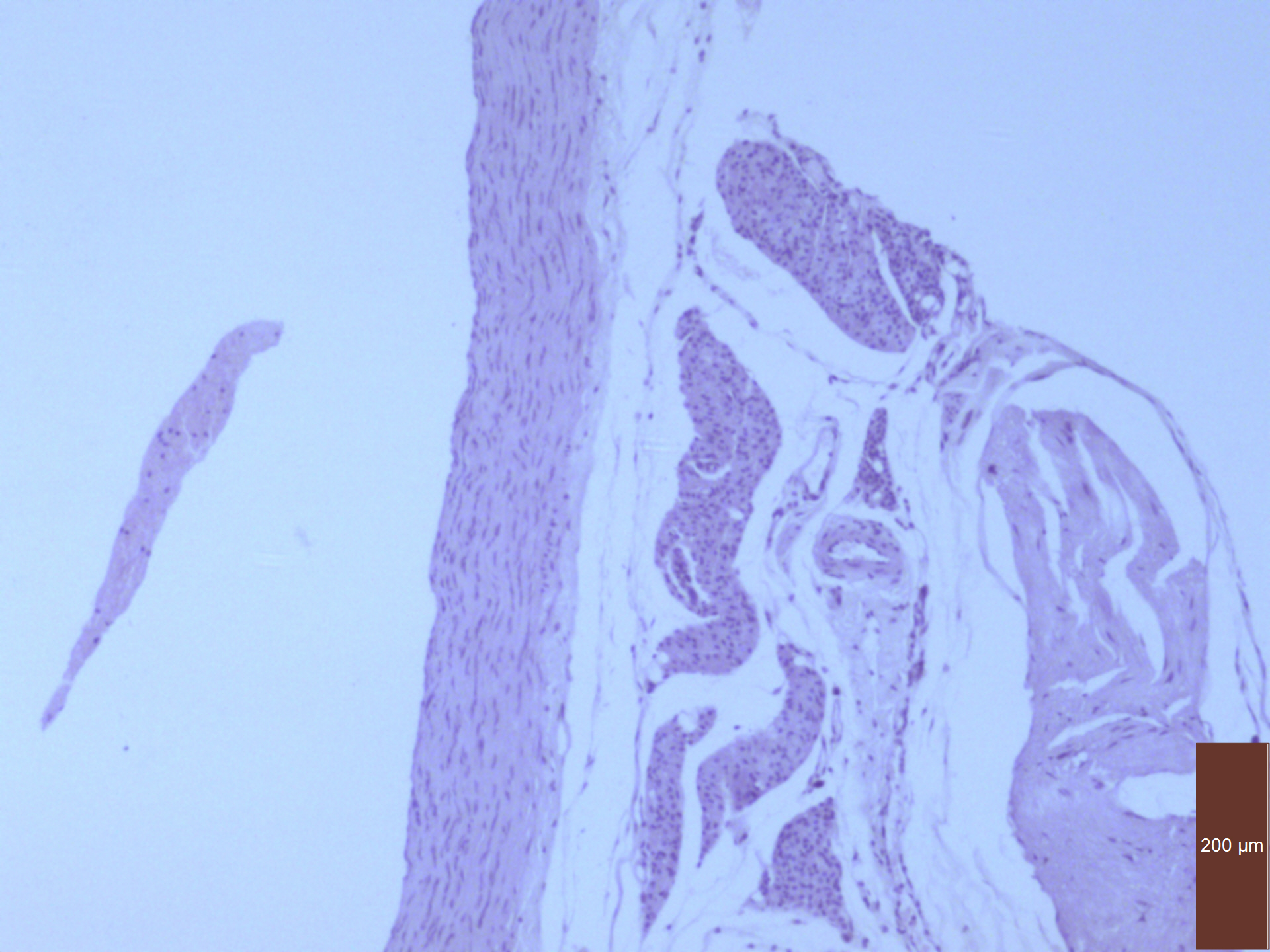

Supplement: Supplementary Materials — All data that involved in this manuscript have been uploaded. [file 5790275.f1.zip › 5790275.f1/control group-100.jpg]

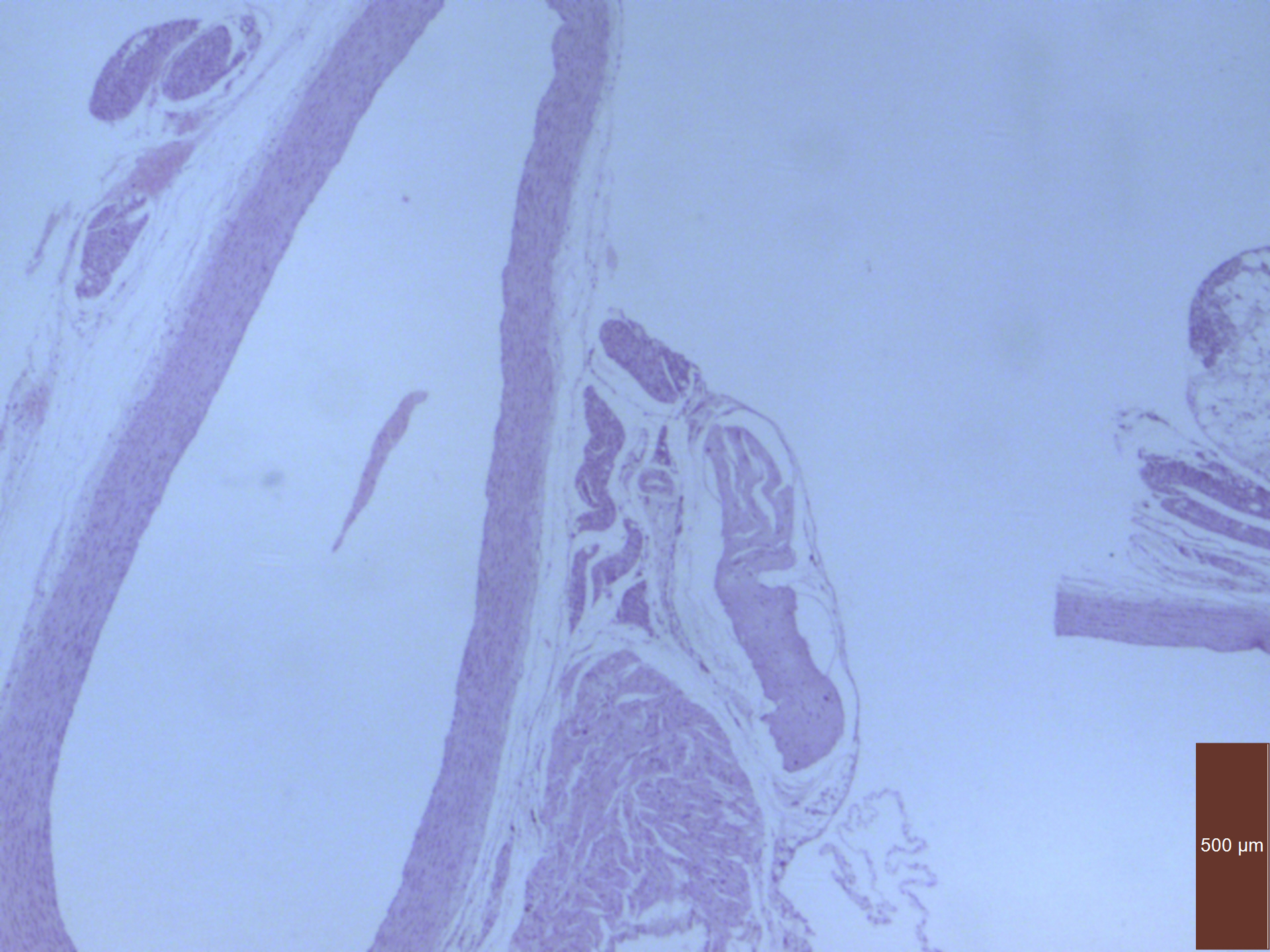

Supplement: Supplementary Materials — All data that involved in this manuscript have been uploaded. [file 5790275.f1.zip › 5790275.f1/control group-40.jpg]

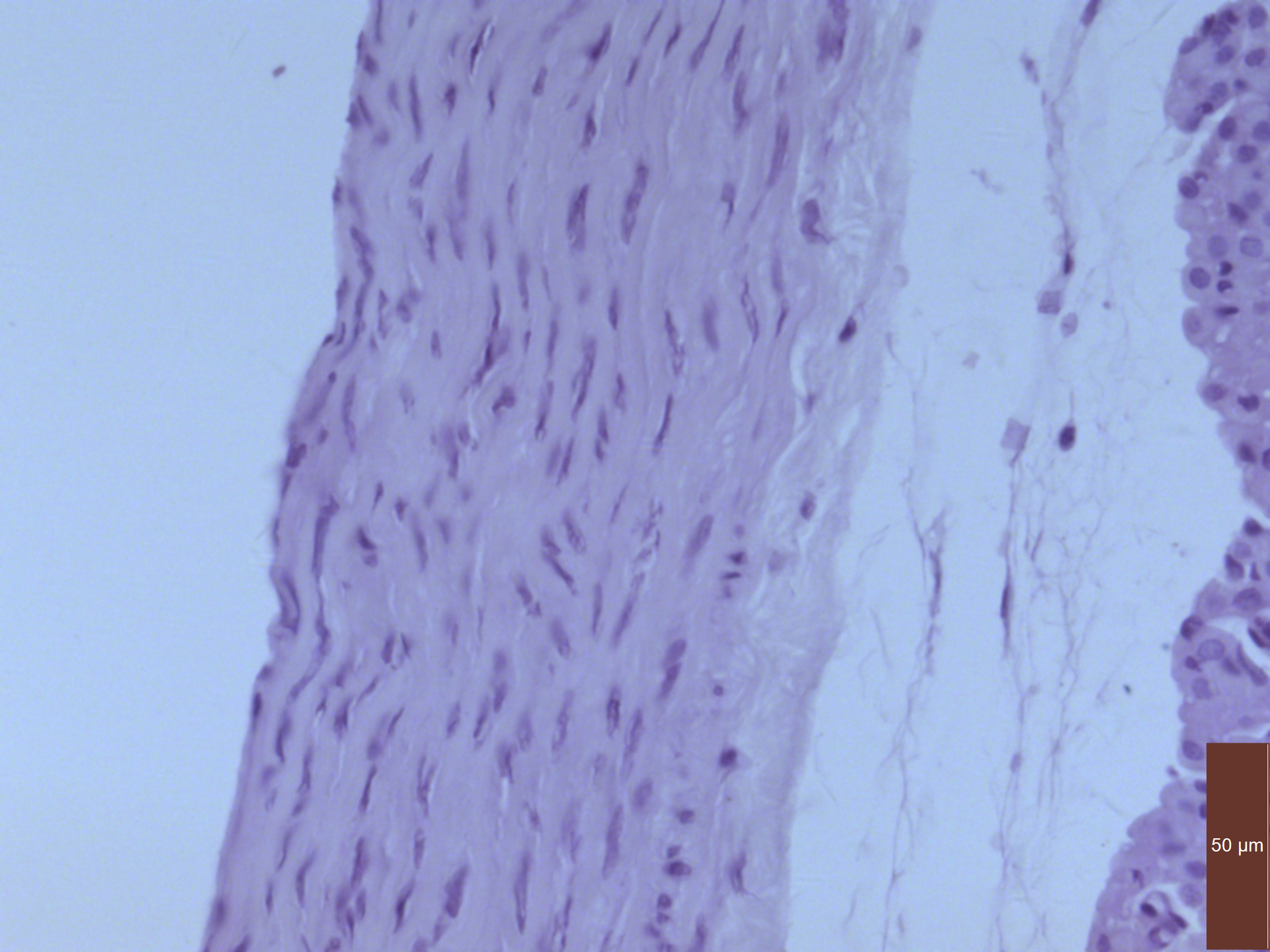

Supplement: Supplementary Materials — All data that involved in this manuscript have been uploaded. [file 5790275.f1.zip › 5790275.f1/control group-400.jpg]

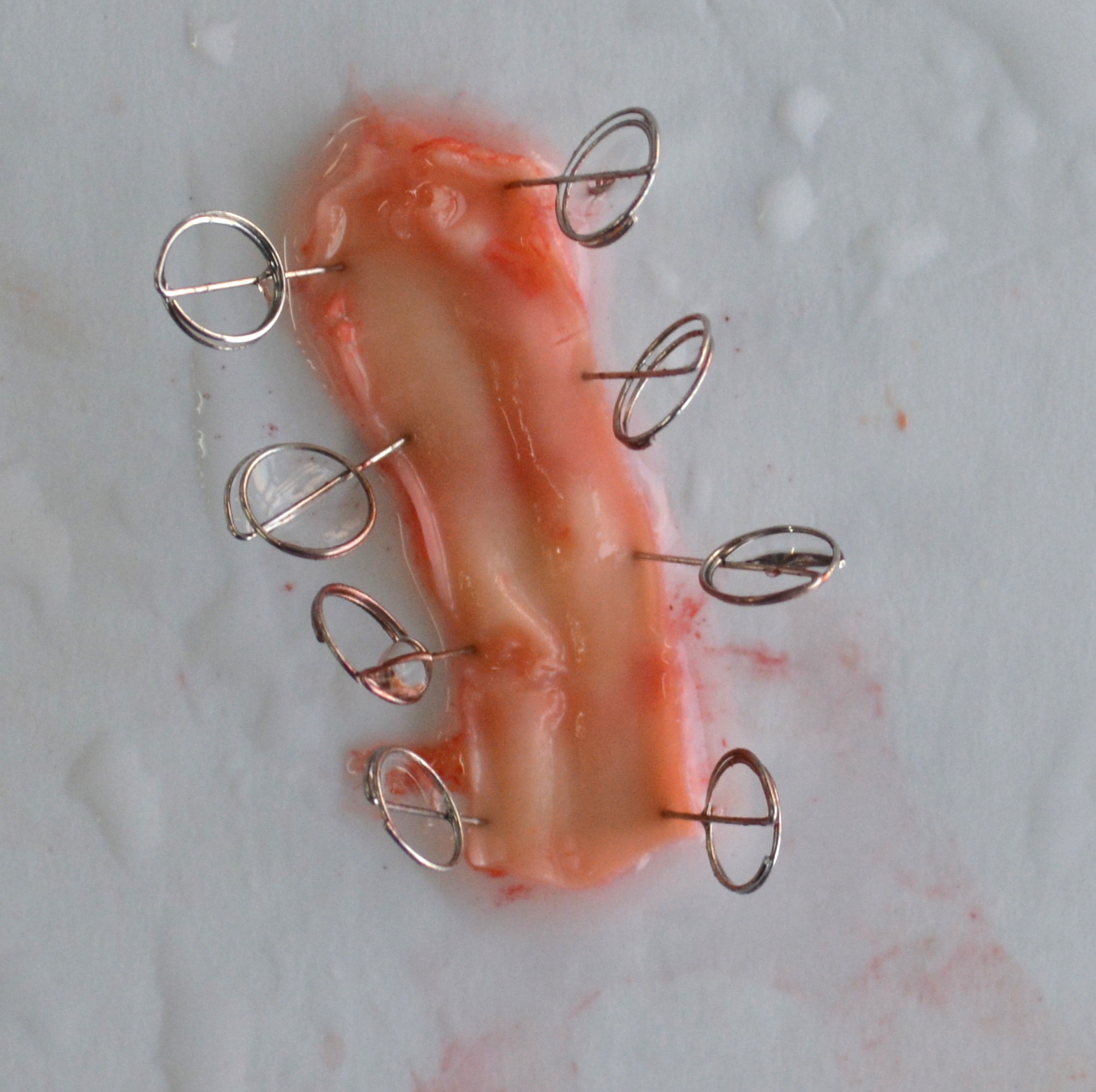

Supplement: Supplementary Materials — All data that involved in this manuscript have been uploaded. [file 5790275.f1.zip › 5790275.f1/control.JPG]

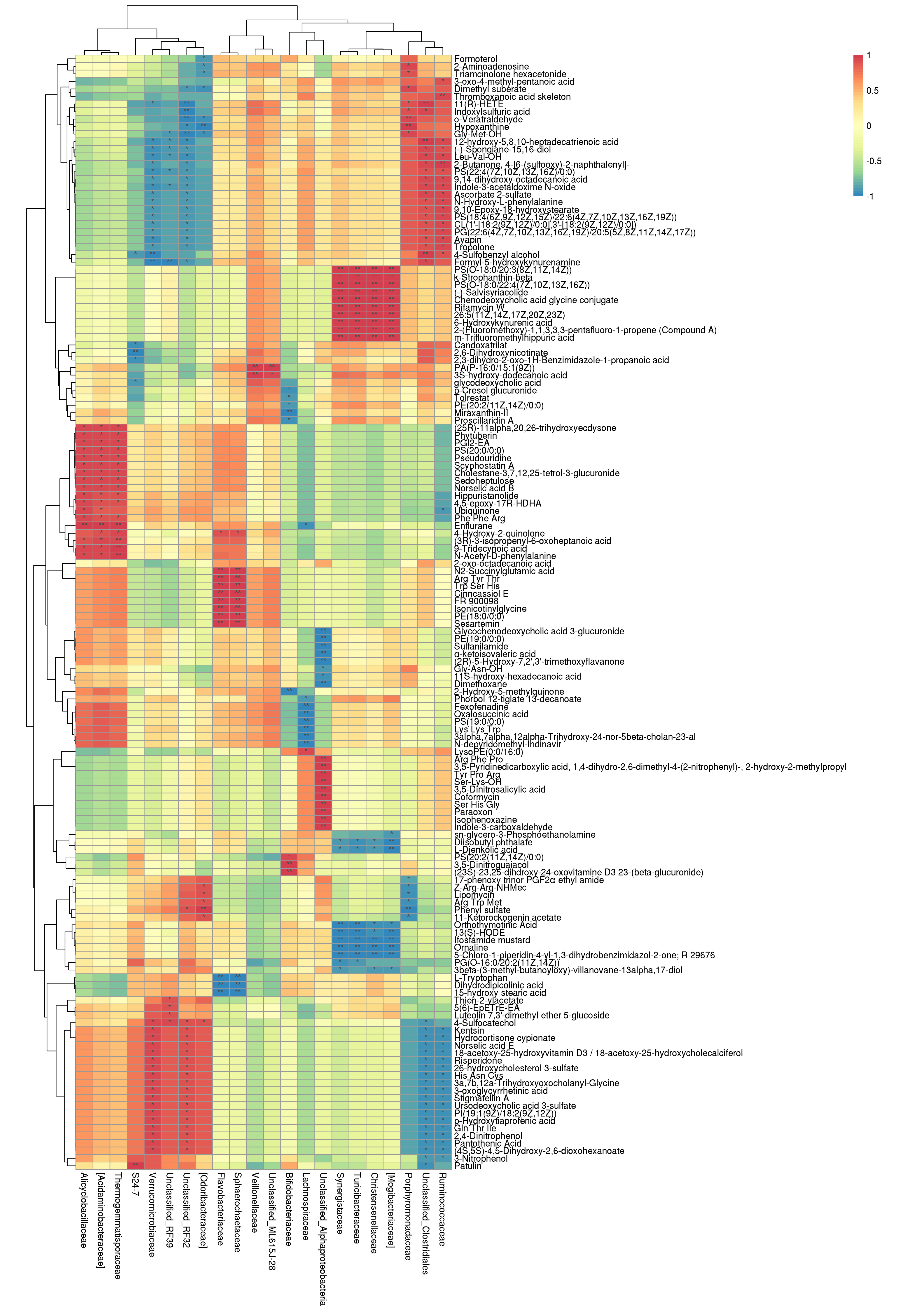

Supplement: Supplementary Materials — All data that involved in this manuscript have been uploaded. [file 5790275.f1.zip › 5790275.f1/corHeatmap_all.png]

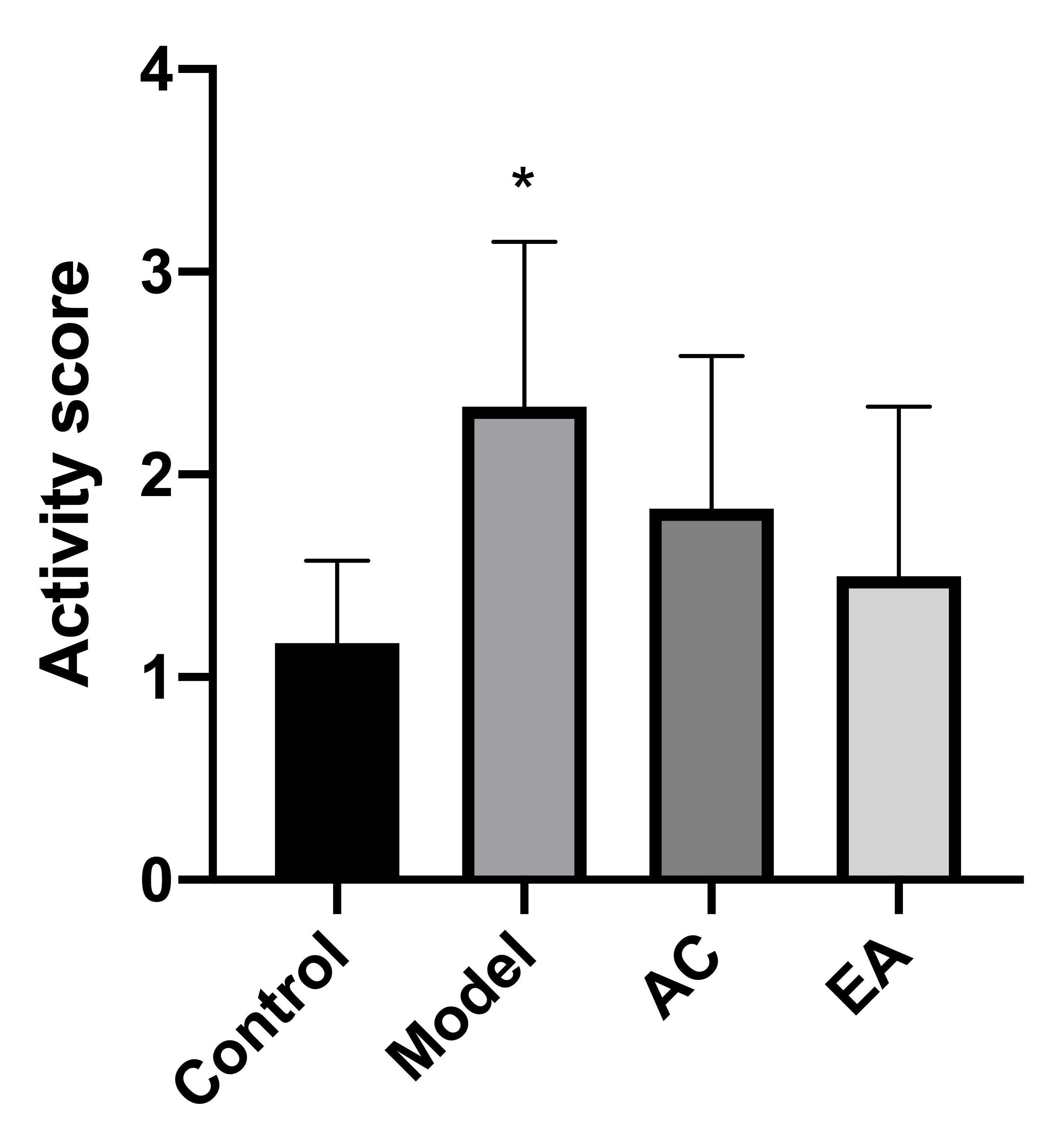

Supplement: Supplementary Materials — All data that involved in this manuscript have been uploaded. [file 5790275.f1.zip › 5790275.f1/Data 1.jpg]

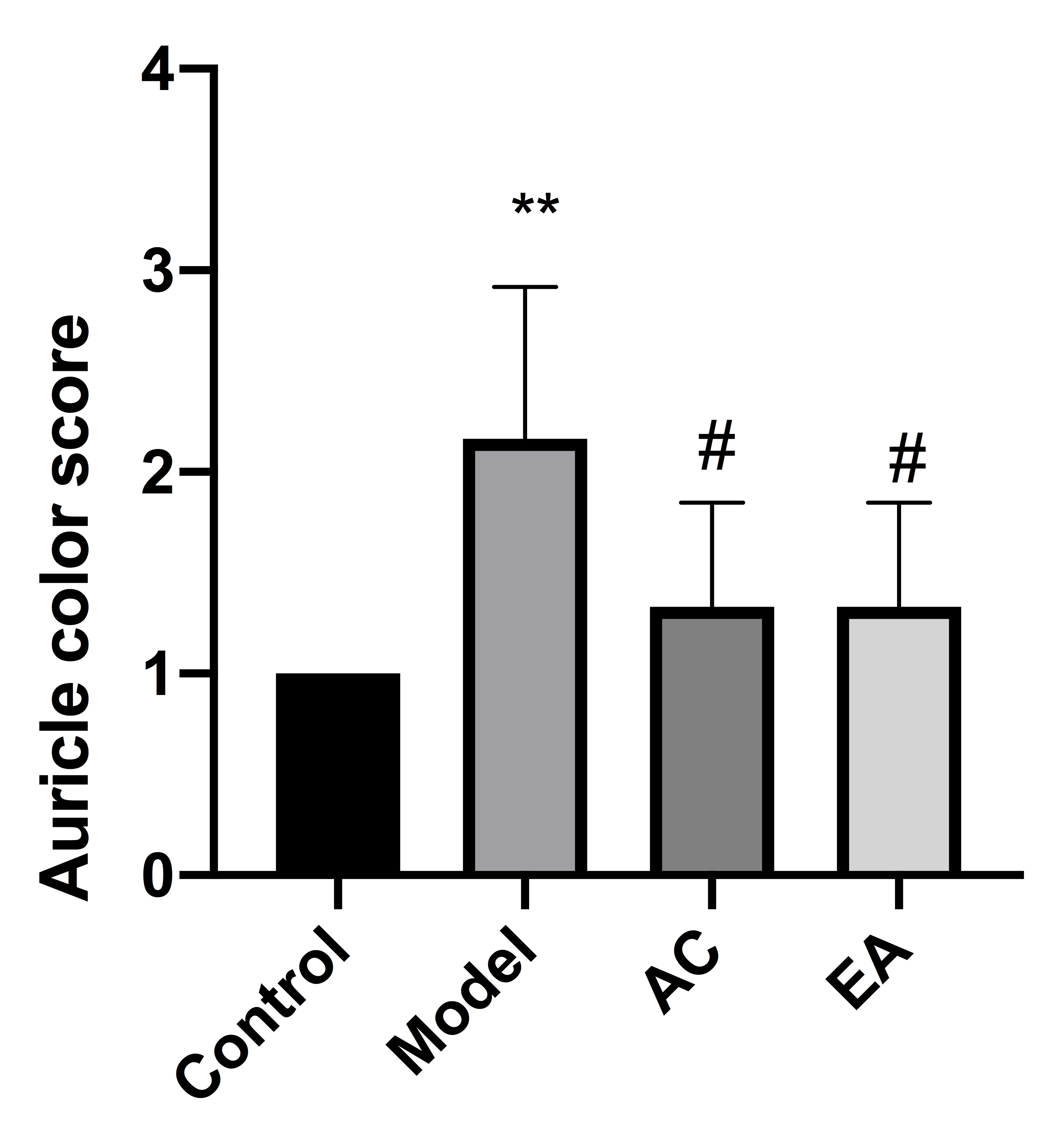

Supplement: Supplementary Materials — All data that involved in this manuscript have been uploaded. [file 5790275.f1.zip › 5790275.f1/Data 2.jpg]

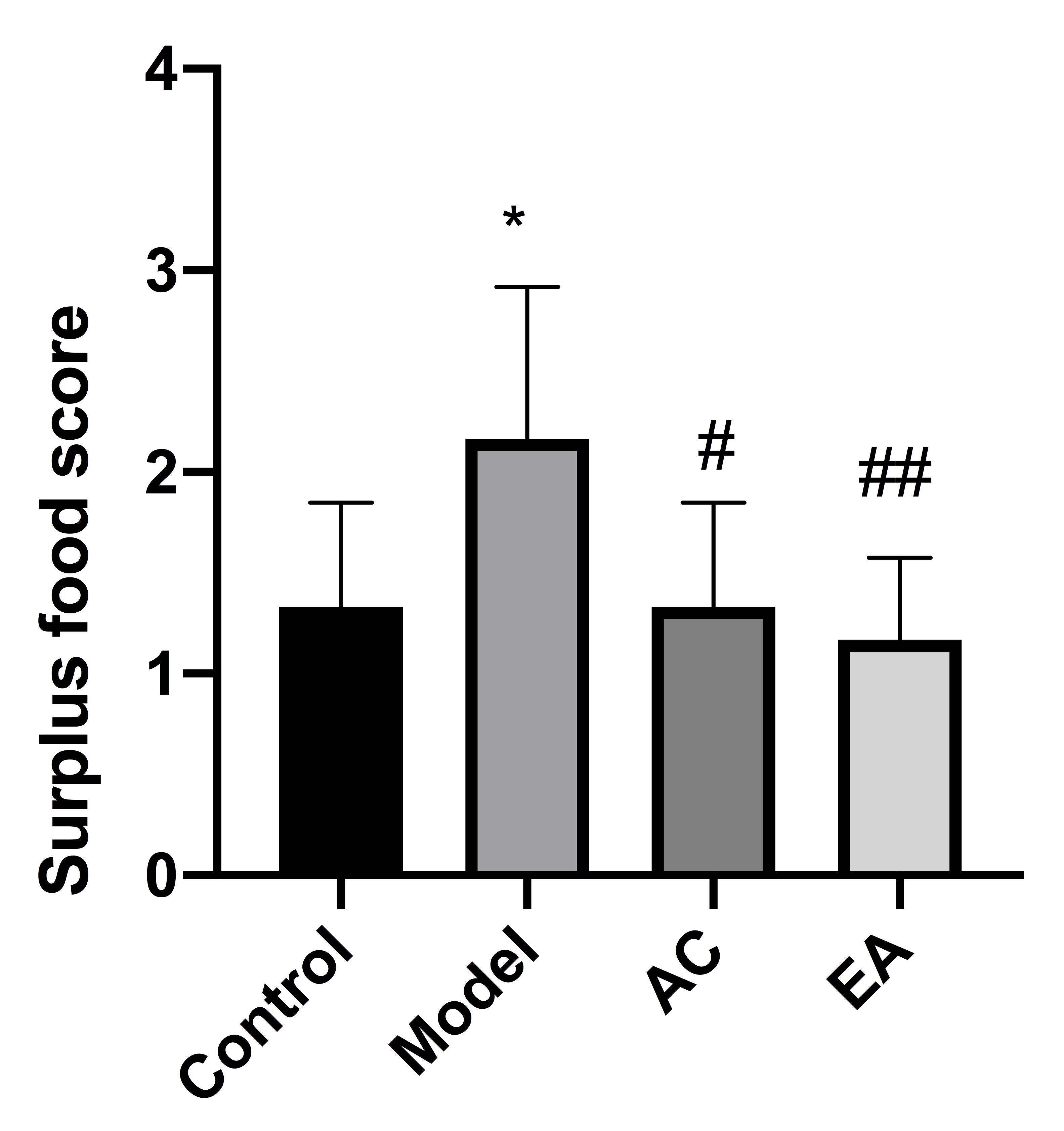

Supplement: Supplementary Materials — All data that involved in this manuscript have been uploaded. [file 5790275.f1.zip › 5790275.f1/Data 3.jpg]

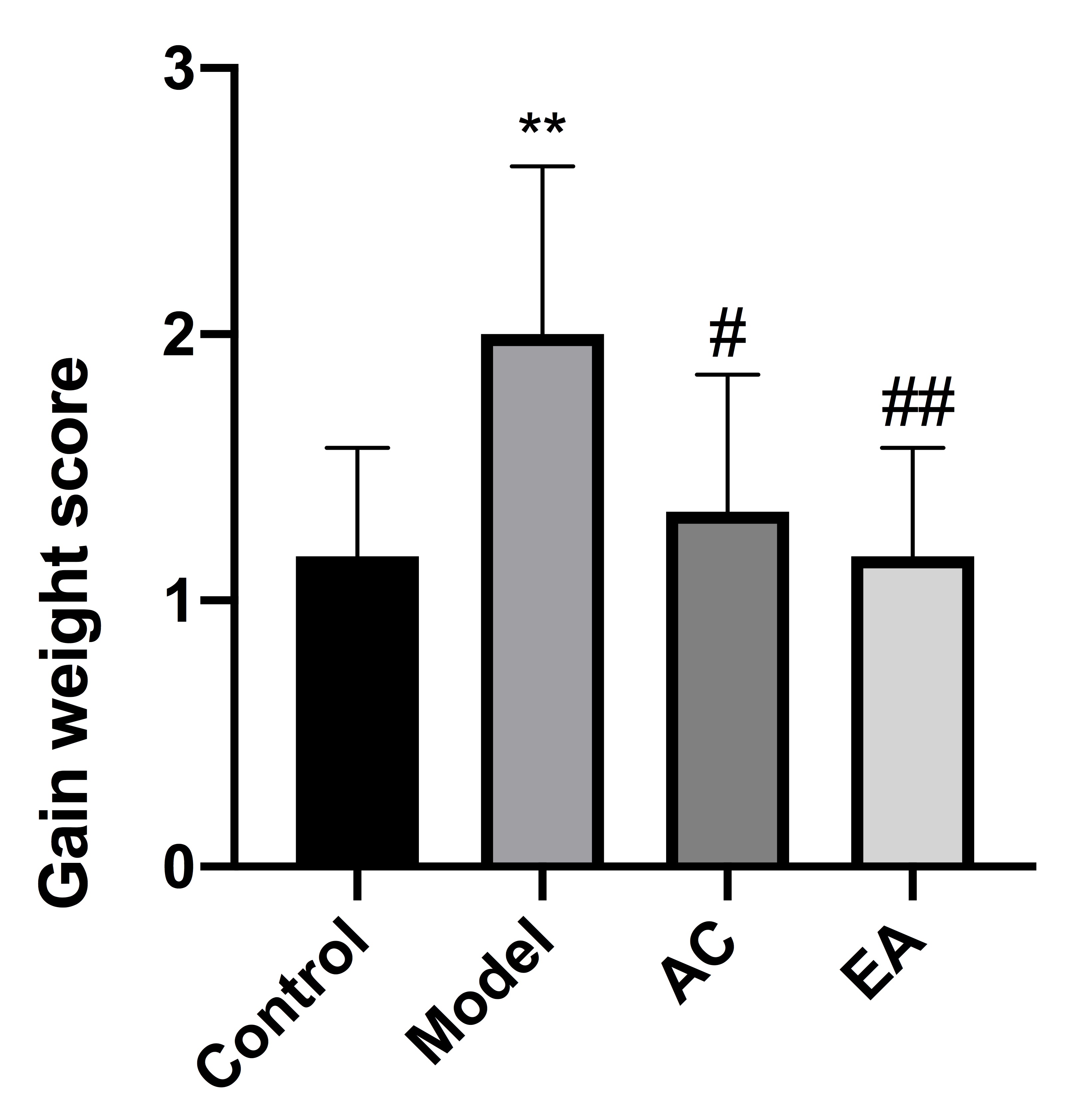

Supplement: Supplementary Materials — All data that involved in this manuscript have been uploaded. [file 5790275.f1.zip › 5790275.f1/Data 4.jpg]

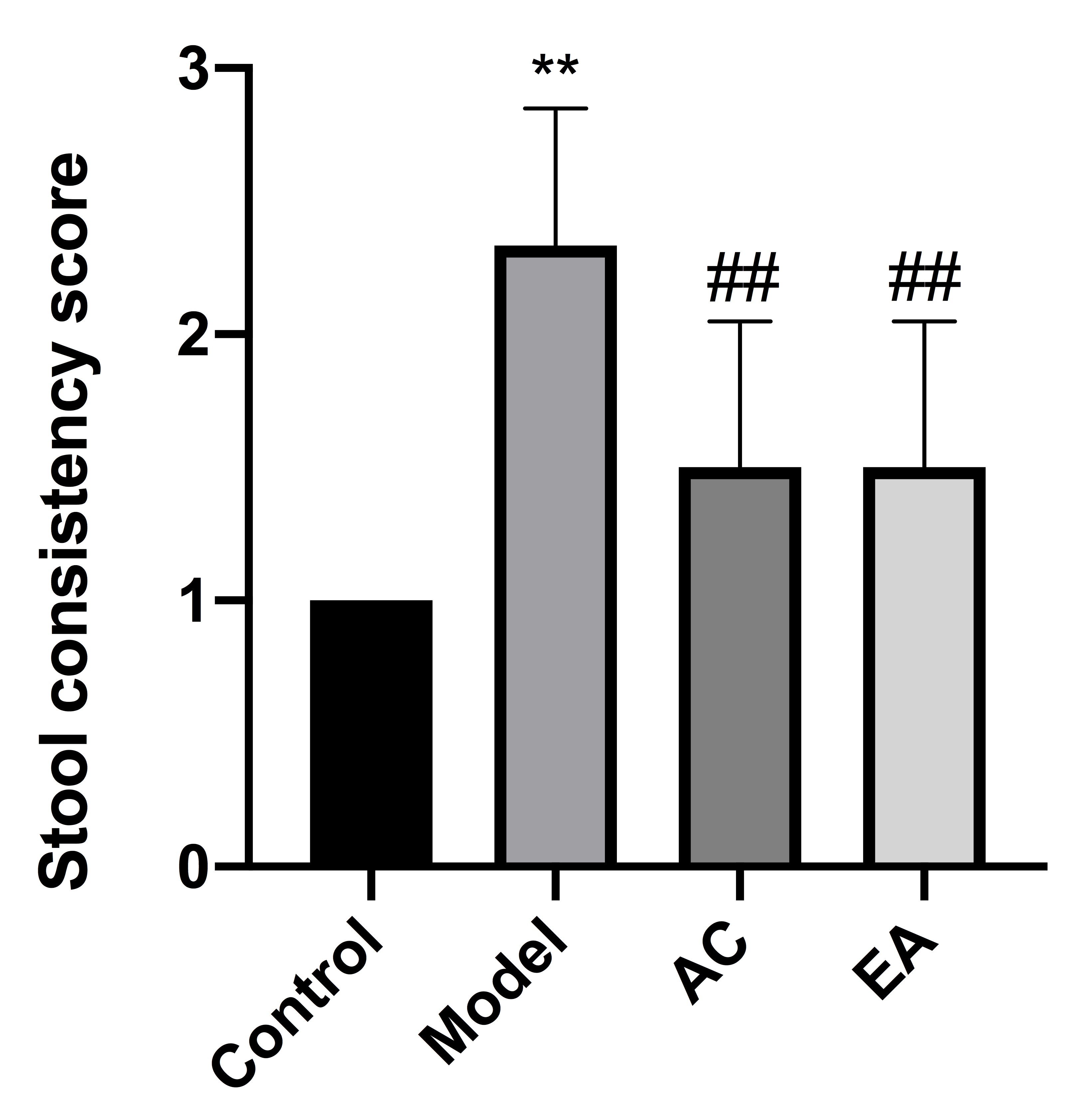

Supplement: Supplementary Materials — All data that involved in this manuscript have been uploaded. [file 5790275.f1.zip › 5790275.f1/Data 5.jpg]

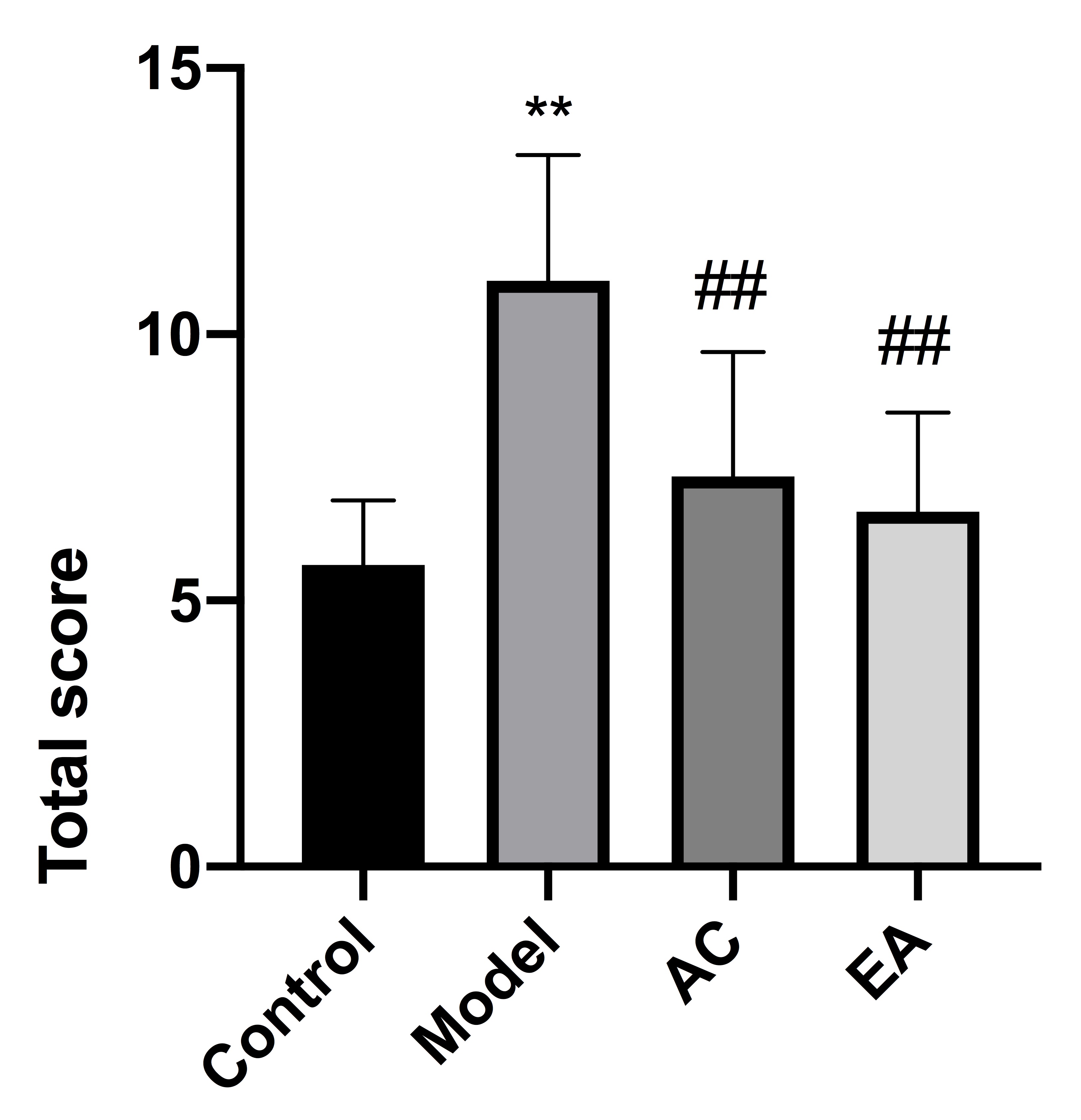

Supplement: Supplementary Materials — All data that involved in this manuscript have been uploaded. [file 5790275.f1.zip › 5790275.f1/Data 6.jpg]

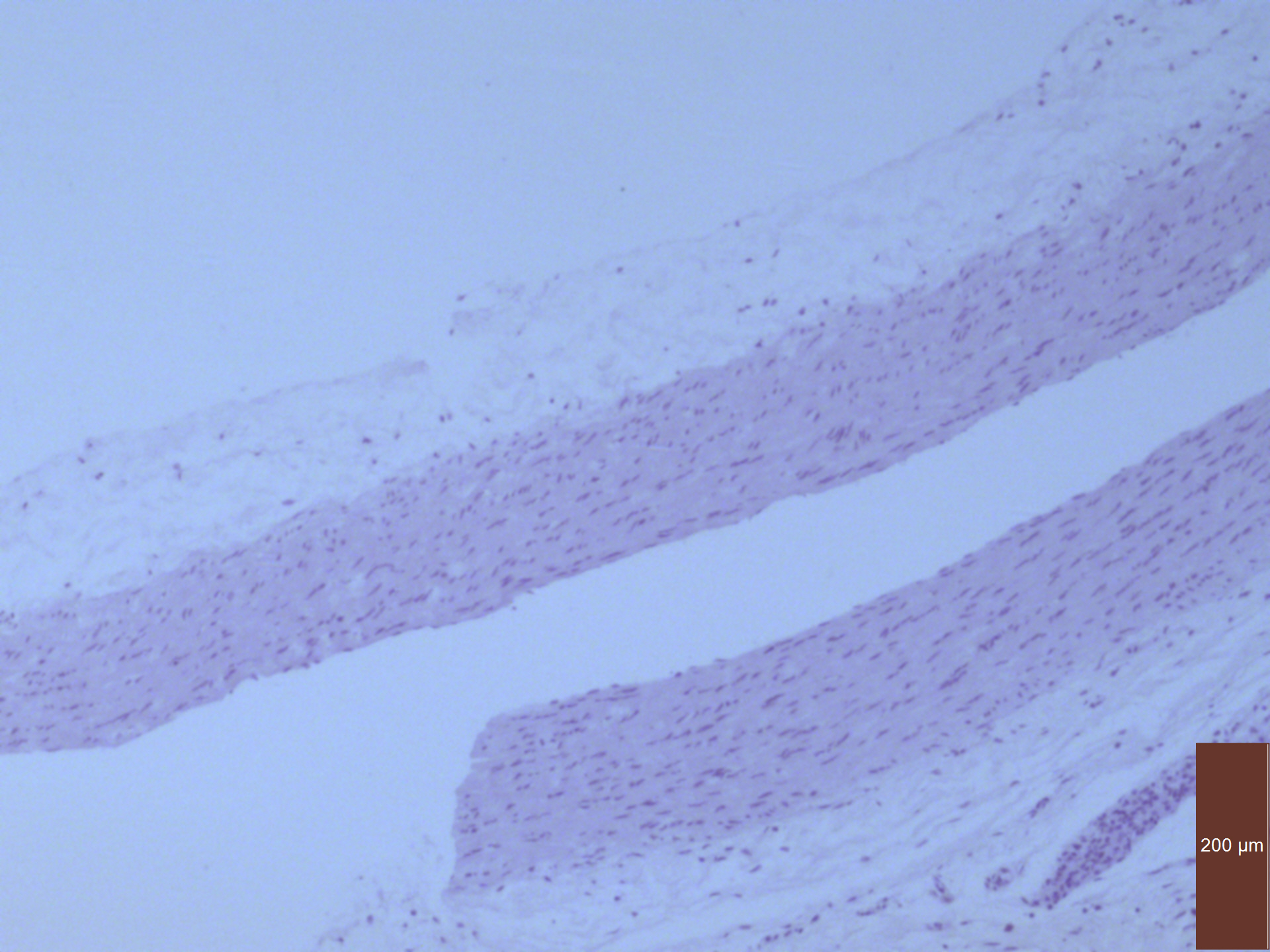

Supplement: Supplementary Materials — All data that involved in this manuscript have been uploaded. [file 5790275.f1.zip › 5790275.f1/EA group-100.jpg]

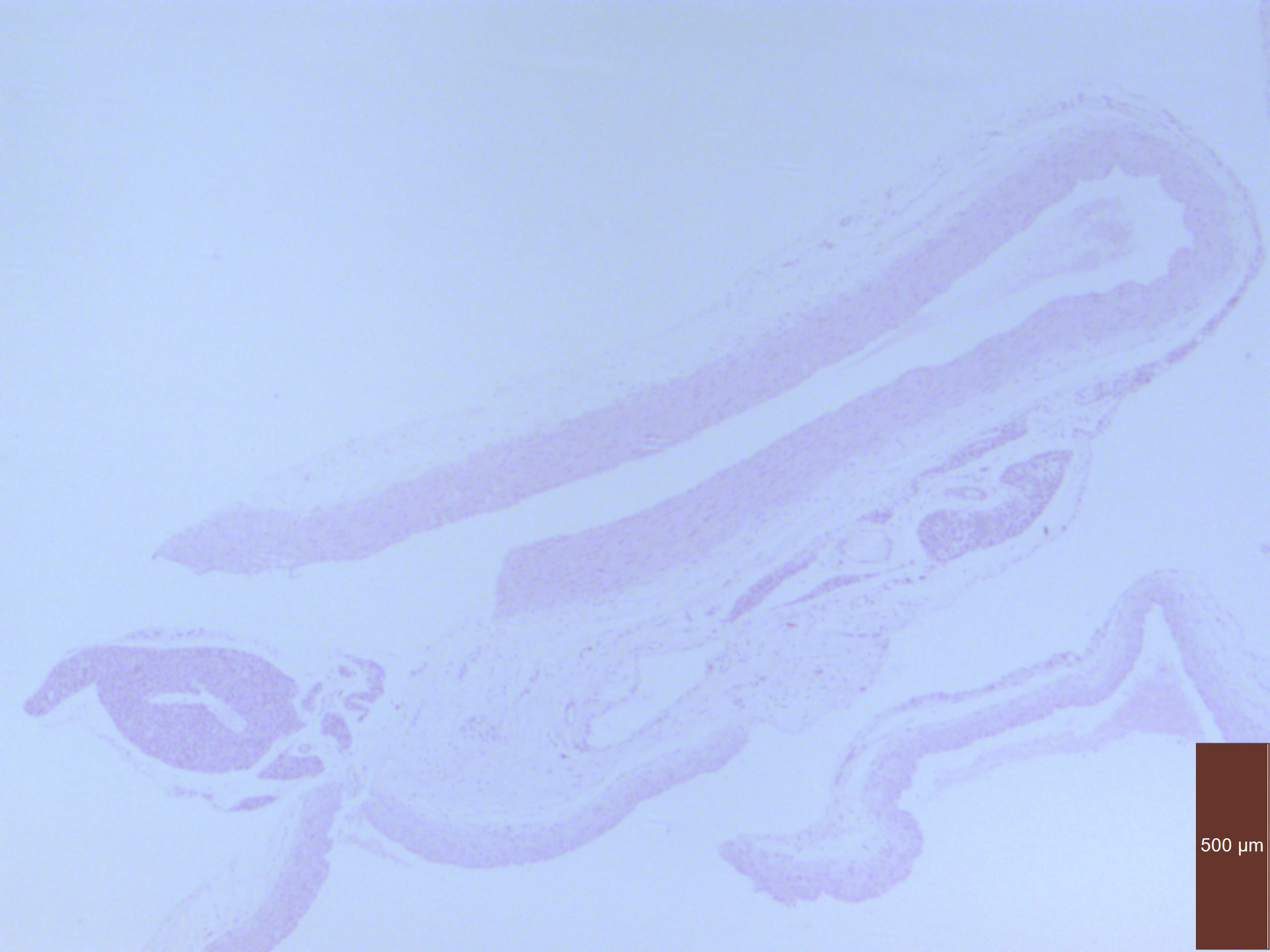

Supplement: Supplementary Materials — All data that involved in this manuscript have been uploaded. [file 5790275.f1.zip › 5790275.f1/EA group-40.jpg]

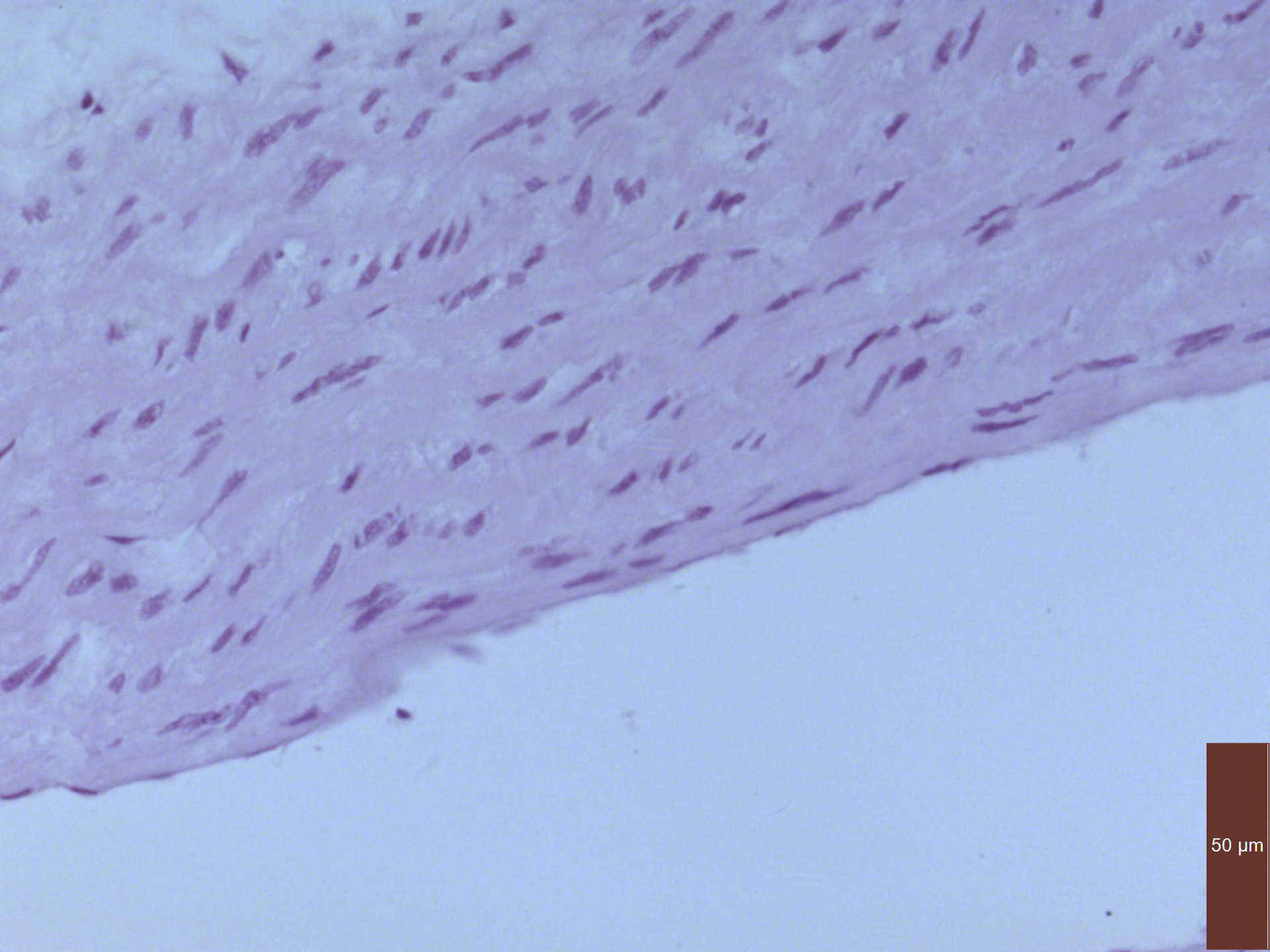

Supplement: Supplementary Materials — All data that involved in this manuscript have been uploaded. [file 5790275.f1.zip › 5790275.f1/EA group-400.jpg]

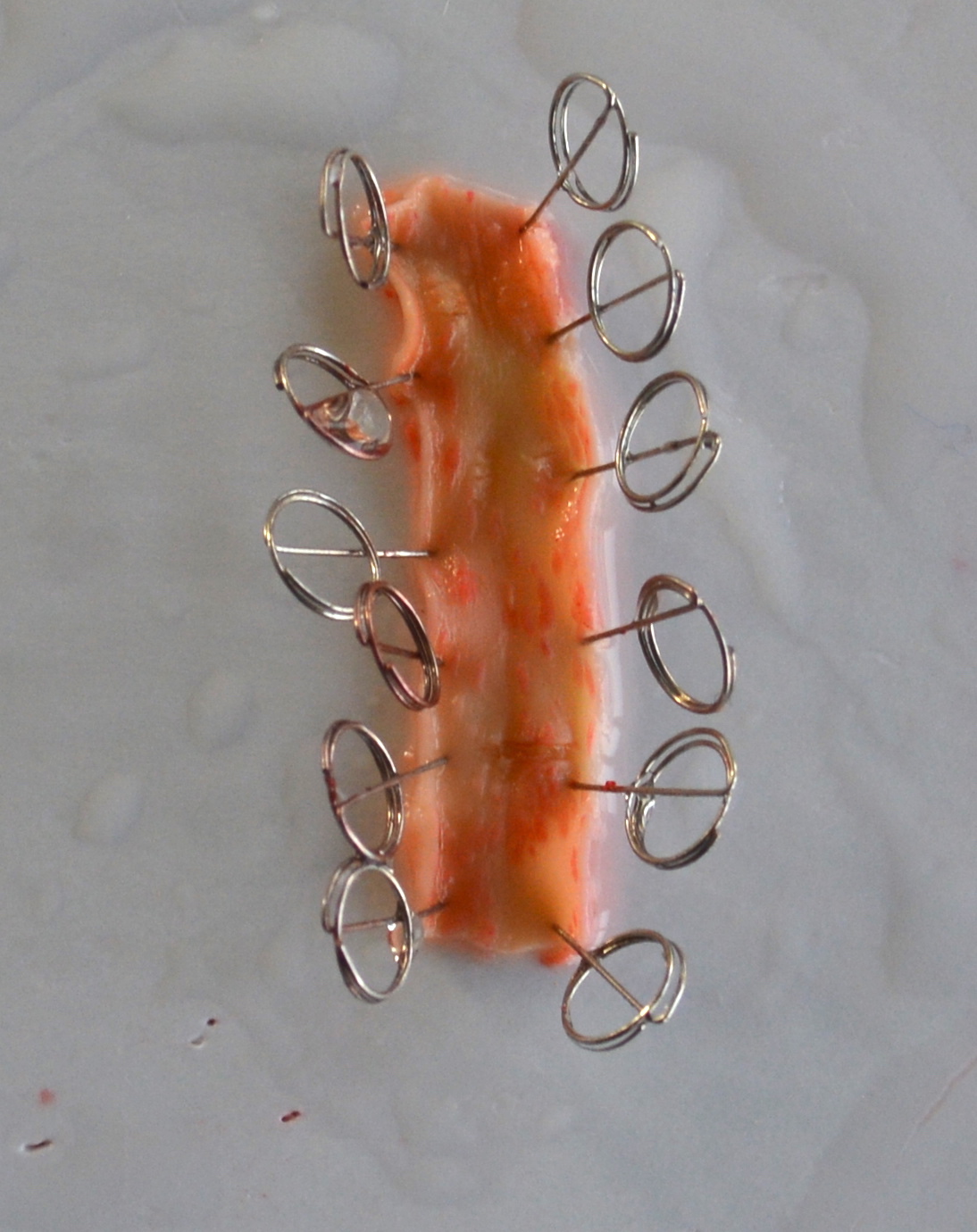

Supplement: Supplementary Materials — All data that involved in this manuscript have been uploaded. [file 5790275.f1.zip › 5790275.f1/EA.JPG]

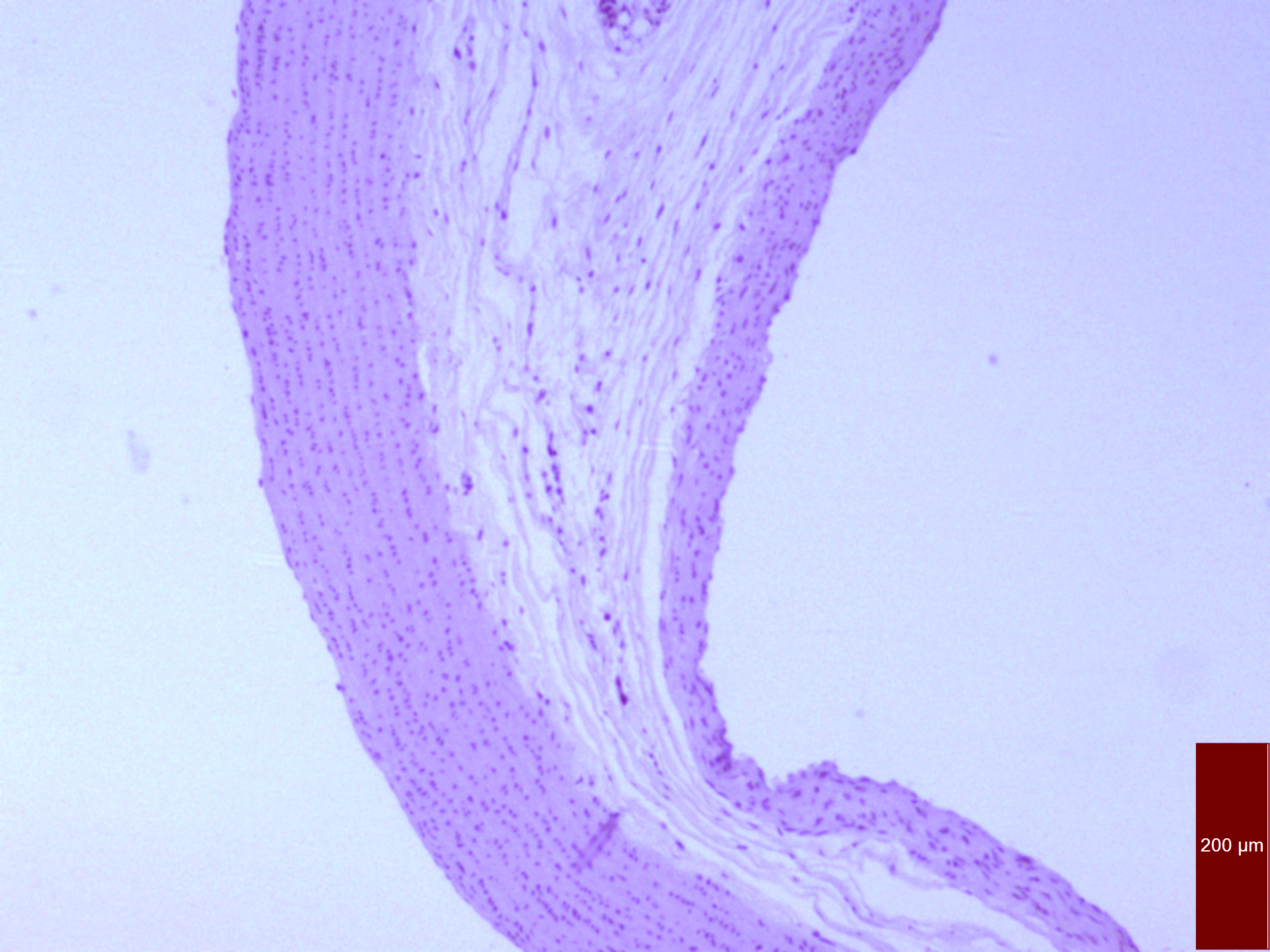

Supplement: Supplementary Materials — All data that involved in this manuscript have been uploaded. [file 5790275.f1.zip › 5790275.f1/model group-100.jpg]

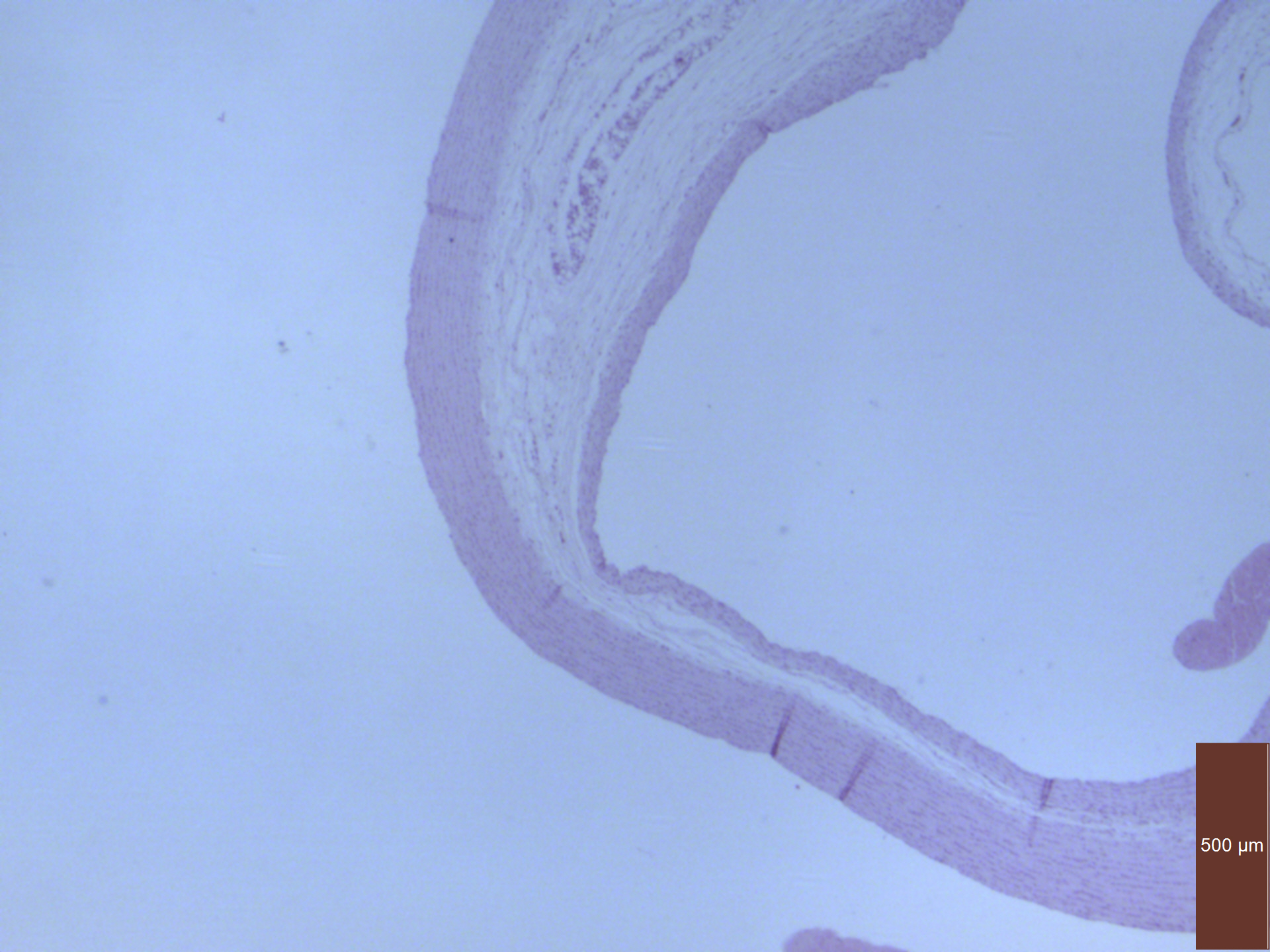

Supplement: Supplementary Materials — All data that involved in this manuscript have been uploaded. [file 5790275.f1.zip › 5790275.f1/model group-40.jpg]

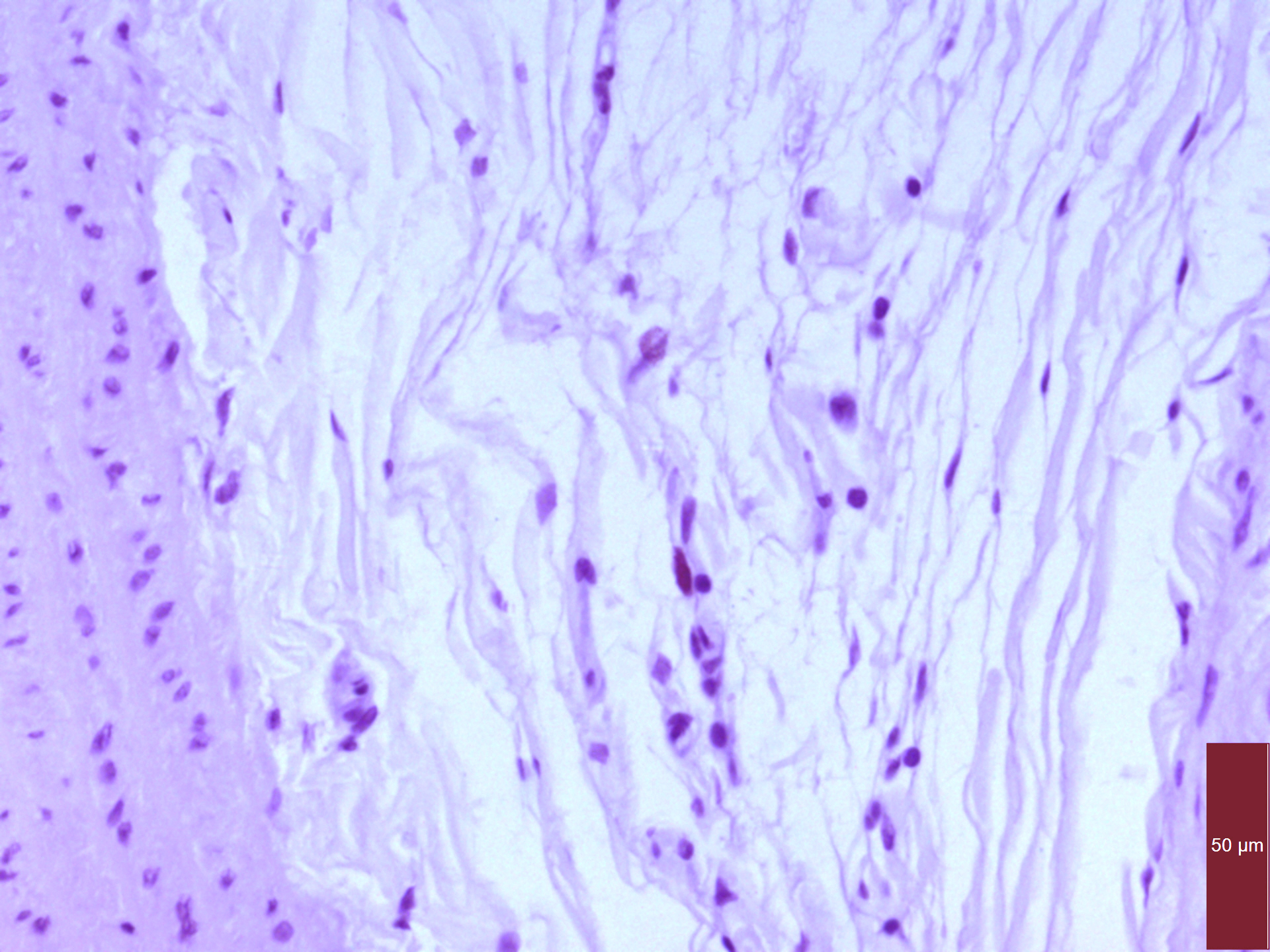

Supplement: Supplementary Materials — All data that involved in this manuscript have been uploaded. [file 5790275.f1.zip › 5790275.f1/model group-400.jpg]

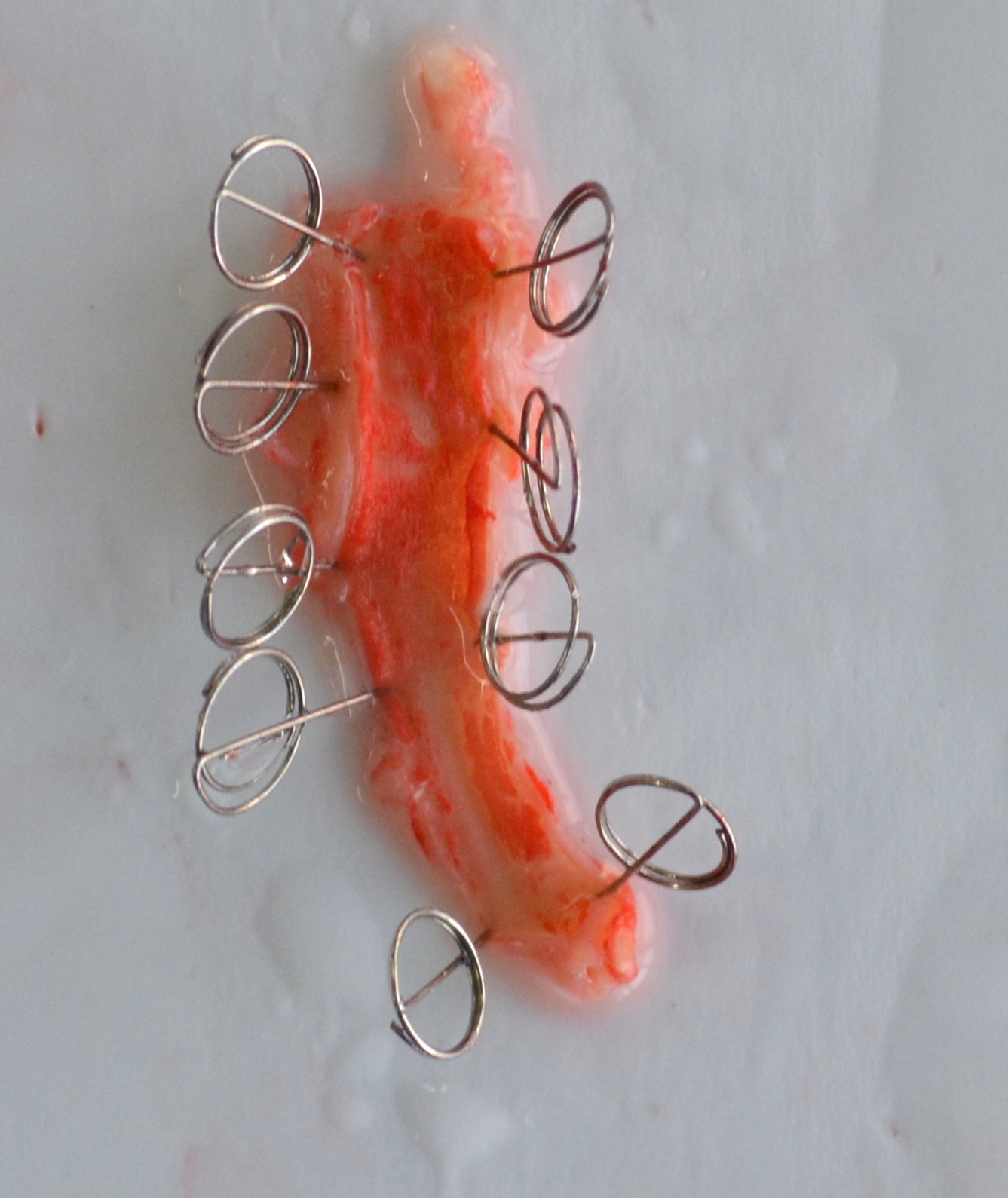

Supplement: Supplementary Materials — All data that involved in this manuscript have been uploaded. [file 5790275.f1.zip › 5790275.f1/model.JPG]

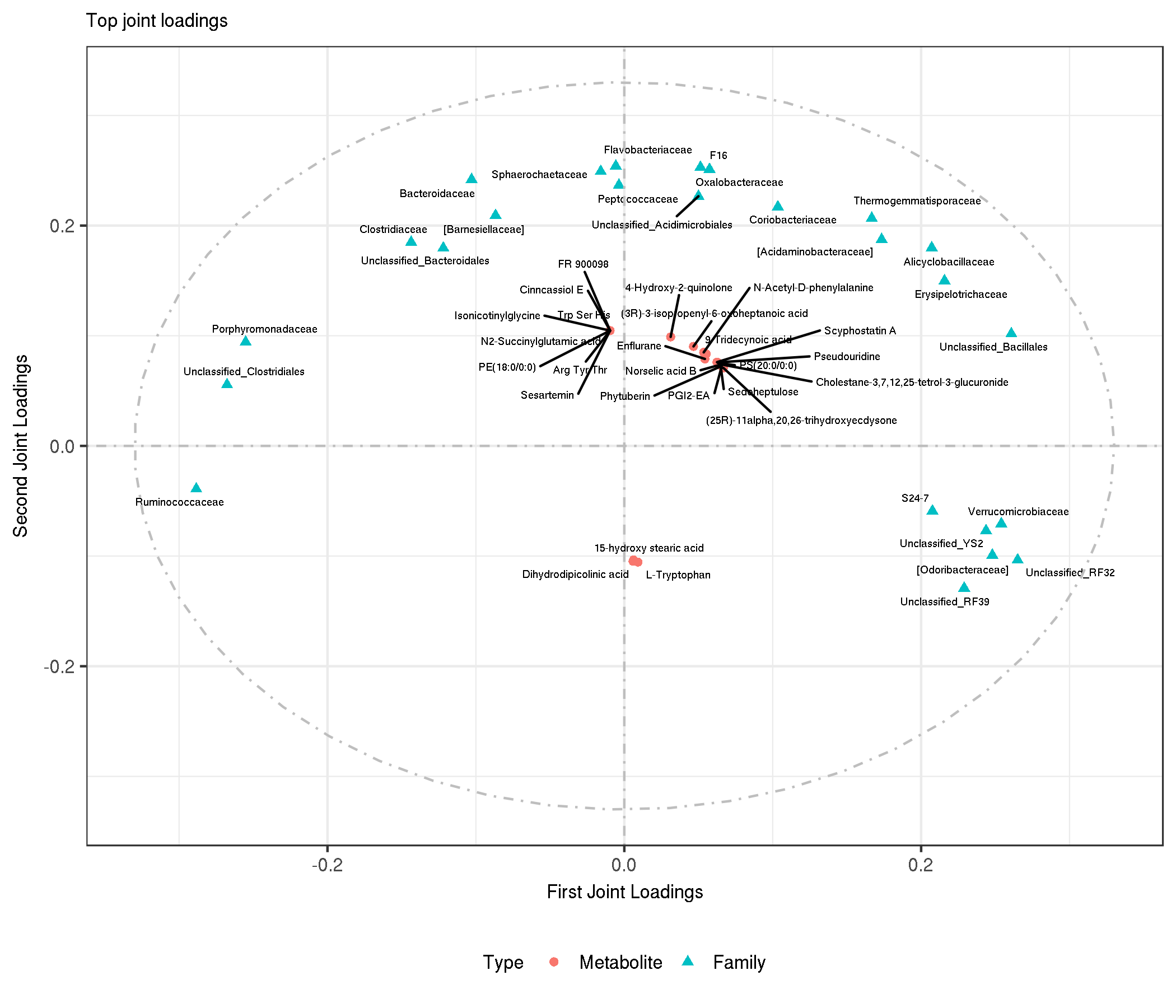

Supplement: Supplementary Materials — All data that involved in this manuscript have been uploaded. [file 5790275.f1.zip › 5790275.f1/top25_loading_element.png]
